# More practical differentially private publication of key statistics in GWAS

**DOI:** 10.1093/bioadv/vbab004

**Published:** 2021-05-18

**Authors:** Akito Yamamoto, Tetsuo Shibuya

**Affiliations:** Division of Medical Data Informatics, Human Genome Center, The Institute of Medical Science, The University of Tokyo, Tokyo 108-8639, Japan

## Abstract

**Motivation:** Analyses of datasets that contain personal genomic information are very important for revealing associations between diseases and genomes. Genome-wide association studies, which are large-scale genetic statistical analyses, often involve tests with contingency tables. However, if the statistics obtained by these tests are made public as they are, sensitive information of individuals could be leaked. Existing studies have proposed privacy-preserving methods for statistics in the χ^2^ test with a 3 × 2 contingency table, but they do not cover all the tests used in association studies. In addition, existing methods for releasing differentially private *P*-values are not practical.

**Results:** In this work, we propose methods for releasing statistics in the χ^2^ test, the Fisher’s exact test and the Cochran–Armitage’s trend test while preserving both personal privacy and utility. Our methods for releasing *P*-values are the first to achieve practicality under the concept of differential privacy by considering their base 10 logarithms. We make theoretical guarantees by showing the sensitivity of the above statistics. From our experimental results, we evaluate the utility of the proposed methods and show appropriate thresholds with high accuracy for using the private statistics in actual tests.

**Availability and implementation:**

A python implementation of our experiments is available at https://github.com/ay0408/DP-statistics-GWAS.

**Supplementary information:**

Supplementary data are available at *Bioinformatics Advances* online.

## 1 Introduction

In recent years, the number of datasets containing personal genomic information and medical records has grown rapidly, and the analyses of these data have become very important for investigating the links between diseases and genomes (Weber *et al.*, 2009). In particular, genome-wide association studies (GWAS) is a common genetic statistical analysis used to investigate genetic factors of diseases. A typical study examines millions of single-nucleotide polymorphism (SNP) locations in a given patient population for relationships between SNPs and a disease. In association studies such as GWAS, a case-control study with a contingency table is often used, and general test methods include χ^2^ test, the Fisher’s exact test and the Cochran–Armitage’s trend test.

However, if the statistics such as χ^2^-statistics and *P*-values obtained from these tests are released as they are, sensitive information of individuals could be leaked. For example, Homer *et al.* (2008) showed that it is possible to identify whether individuals with a certain genotype are in a sufficiently complex genomic DNA mixture. Furthermore, Wang *et al.* (2009) demonstrated that individuals can be identified from even a relatively small set of statistics by using correlation statistics between SNPs. After the appearance of these studies, the NIH removed the GWAS aggregate results from the public database, despite the importance of analyses based on *P*-values of statistical tests (Chen and Yang, 2019; Zaykin and Kozbur, 2010). This has become a major hindrance for research on the genetic factors of diseases (Zerhouni and Nabel, 2008).

In this situation, it is very important to find a way to enable the release of GWAS statistics data without compromising the privacy of individuals, and the concept of differential privacy (Dwork, 2006) might be useful for this purpose. Differential privacy is a framework for quantifying the extent to which the privacy of individuals in a database is guaranteed when releasing useful information, such as statistics. It aims to achieve strong privacy guarantees by considering situations in which it is almost impossible to distinguish whether the database contains a particular individual, regardless of the information held by an adversary. This concept has been incorporated into deep learning techniques (Abadi *et al.*, 2016) and applied to the sharing of medical data (Raisaro *et al.*, 2019), for example, and it is expected to be further used to protect genomic data in the future.

Fienberg *et al.* (2011) proposed a new method for releasing some private data in GWAS using the concept of differential privacy. This method focused on the sensitivity of the statistical function and applied the privacy protection mechanism presented by Dwork *et al.* (2006). The article introduced a privacy-preserving methodology for the release of the averaged minor allele frequencies (MAF) of the case and those of the control in GWAS, and *ϵ*-differentially private $\chi^{2}$-statistics and *P*-values based on a 3 × 2 contingency table. However, the method for releasing *P*-values is less practical. Moreover, there are a few other statistics that could be made public, for example, $\chi^{2}$-statistics and *P*-values based on a 2 × 2 contingency table (Dickhaus *et al.*, 2012; Matthews *et al.*, 2008), *P*-values obtained from Fisher’s exact test (Fisher, 1935), and the statistics from the Cochran–Armitage’s trend test (Armitage, 1955).

$\chi^{2}$ -statistics and *P*-values in the *χ*^2^ test based on a 3 × 2 contingency table are mainly used to compare genotype frequencies between the case and the control, whereas those based on a 2 × 2 contingency table are often used to compare allele frequencies. The Fisher’s exact test is commonly used in place of the χ^2^ test when the entries of a contingency table are small. The Cochran–Armitage’s trend test corresponds to the logical regression score test and is used to test the additive genetic model (Zeng *et al.*, 2015). Other statistical tests used in GWAS include Yate’s correction for continuity (Yates, 1934) and McNemar’s test utilized for transmission disequilibrium test (Spielman *et al.*, 1993), for example, but this paper focuses on the above three methods, which are the most common methods using contingency tables.

In this work, we propose methods to make the statistics obtained from the above three statistical tests public while preserving the privacy of individuals. Our privacy assurances use the concept of differential privacy, similar to the approach of Fienberg *et al.* (2011). Firstly, based on their work, we show how to release *P*-values in the χ^2^ test using a 3 × 2 contingency table while ensuring utility. Then, we present methods for releasing $\chi^{2}$-statistics and *P*-values in the χ^2^ test based on a 2 × 2 contingency table, which is used to test whether the allele frequencies differ between the case and the control. Secondly, we describe methods for releasing *P*-values obtained from the Fisher’s exact test. Finally, we show how to release $\chi^{2}$-statistics and *P*-values obtained from the Cochran–Armitage’s trend test to check whether there is a linear trend in the ratio of each row in a 3 × 2 contingency table. This test method is often used for genotype frequency comparisons. Subsequently, we evaluate the utility of these methods by experiments. From the results, we show that the methods for releasing $\chi^{2}$-statistics in the χ^2^ test and the Cochran–Armitage’s trend test are practical. As for the methods for the Fisher’s exact test, they are shown to be useful when the total number of individuals included in a contingency table is small. Regarding the revelation of the private *P*-values, which has been considered difficult in previous studies, we show that it is possible to obtain utility by considering their base 10 logarithms. In addition, we describe how to use these private statistics and set appropriate thresholds with high accuracy in actual tests.

In Section 2, we present methods for releasing *ϵ*-differentially private statistics for each test. In Section 3, we evaluate their utility based on a simulation study and show appropriate thresholds of the private statistics. We summarize our study with future work in Section 4.

In the supplement, we discuss details of statistical tests and differential privacy, as well as recent researches on GWAS data. It also includes more detailed proofs of our methods.

## 2 Methods

A typical GWAS examines the relationship between SNPs and a disease status of individuals. One of the simplest association analyses used in the examinations is the case-control test with a contingency table. 3 × 2 and 2 × 2 contingency tables are used to compare genotype frequencies and allele frequencies, respectively. The disease status is often represented by a binary phenotype, which takes values 0 and 1. In a 3 × 2 table, the genotype takes values 0, 1 and 2, representing the number of minor alleles. In a 2 × 2 table, the values 0 and 1 for alleles refer to the major allele and the minor allele, respectively. The value in each cell (*i*, *j*) of the contingency table is the number of individuals with genotype or allele *i* and disease status *j*. In GWAS, the number of the case and that of the control are generally set close to each other, so we assume that the total number of individuals is denoted by *N*, and that there are N/2 cases and N/2 controls. Since GWAS usually considers thousands to millions of individuals, we set N≥100 for sake of simplicity in this work. We also assume that all margins of contingency tables are positive, because GWAS generally removes SNPs with an MAF smaller than 0.05. Based on the above assumptions, we calculate the sensitivity of statistics in the χ^2^ test, the Fisher’s exact test and the Cochran–Armitage’s trend test. Then, we show *ϵ*-differentially private algorithms for releasing those statistics. The definition of *ϵ*-differentially privacy (Dwork, 2006) is as follows:

Definition 1. *A randomized mechanism M is ϵ-differentially private if, for all datasets D and Dʹ, which differ in only one individual and any S*⊂*range(M)*,

$$\text{Pr}[M(D)\in S]\leq e^{\epsilon }\cdot \text{Pr}[M(D\prime )\in S].$$

To satisfy the definition of *ϵ*-differential privacy, we consider the *sensitivity* of a function. The following is the definition of the *sensitivity*.

Definition 2. *Let*$D^{N}$*be the collection of all datasets with N individuals, the sensitivity of a function*$f:D^{N}\to R^{d}$*is*

$$\Delta f={\underset{D,D\prime }{\max}}_{}||f(D)-f(D\prime )|{|}_{1},$$

*where*$D,D\prime \in D^{N}$*differ in a single individual*.

For a statistic *f*(*D*) obtained from the original dataset *D*, releasing f(D)+b satisfies *ϵ*-differential privacy when *b* is random noise derived from a Laplace distribution with mean 0 and scale $\frac{\Delta f}{\epsilon }$ (Dwork *et al.*, 2006). This releasing method is often called as the Laplace mechanism. When using this mechanism, private statistics can be output by simply adding a perturbation to each statistic, so the computational complexity is the same as when the original statistics are released.

### 2.1 *ϵ*-Differentially private statistics for χ^2^ test

Fienberg *et al.* (2011) showed how to release $\chi^{2}$-statistics and *P*-values for a 3 × 2 contingency table used for genotype frequency comparisons. However, when it comes to *P*-values, their method is not practical because the amount of added noise is too large compared to the original *P*-values. In addition, statistical tests in GWAS can also use a 2 × 2 contingency table. In the following, we consider a practical method for releasing *P*-values in the case with a 3 × 2 table and $\chi^{2}$-statistics and *P*-values in the case with a 2 × 2 table.

#### 2.1.1 Case 1: 3 × 2 contingency table

We propose to release the base 10 logarithm of the *P*-values [${\;\text{log}\;}_{10}$(*P*-values)] while preserving privacy. This is because if we try to release the *P*-values themselves, the random noise would be much larger than the original *P*-values and the noise-added statistics, which have become smaller than zero must be rounded to over zero. If we consider the value of $-{\;\text{log}\;}_{10}$(*P*-values), the threshold for the test becomes larger and there is no upper limit to the value. In the following, we will show the sensitivity of the ${\;\text{log}\;}_{10}$(*P*-values) and present the method for releasing that value.

Theorem 1. *The sensitivity of*${\;\text{log}\;}_{10}$*(P-values) obtained from the*$\chi^{2}$*-statistic for genotype frequency comparisons based on a*3×2*contingency table, in which the margins are positive and the number of the case and the control are both*N/2*, is*${\;\text{log}\;}_{10}(e)\cdot \frac{2N}{N+2}$.

Proof. Let *x* be the $\chi^{2}$-statistic obtained from a 3 × 2 contingency table. The *P*-value corresponding to *x* is $e^{-\frac{x}{2}}$, and the base 10 logarithm of the value is $-\frac{x}{2}\cdot {\;\text{log}\;}_{10}(e)$.

From Fienberg *et al.* (2011), the sensitivity of the $\chi^{2}$-statistics is $\frac{4N}{N+2}$. Therefore, the sensitivity of ${\;\text{log}\;}_{10}$(*P*-values) is

$$\left|-\frac{1}{2}\;\text{log}(e)\cdot \frac{4N}{N+2}\right|={\;\text{log}\;}_{10}(e)\cdot \frac{2N}{N+2}.$$

□

In order to release the *ϵ*-differentially private ${\;\text{log}\;}_{10}$(*P*-values), we need to add Laplace noise with scale $\frac{1}{\epsilon }\cdot {\;\text{log}\;}_{10}(e)\cdot \frac{2N}{N+2}$ to the true value. When *N* is sufficiently large, the value of the sensitivity is about 0.87, implying that this method is more practical than considering the *P*-values as they are.

#### 2.1.2 Case 2: 2 × 2 contingency table

Here, we consider the $\chi^{2}$-statistics in tests for allele frequencies using 2 × 2 tables, which are also common tests in association studies using SNPs. Note that when the total number of individuals is *N*, the total number of alleles is 2N because each individual has two alleles.

Theorem 2. *The sensitivity of the*$\chi^{2}$*-statistics for allele frequency comparisons based on a*2×2*contingency table, in which the margins are positive and the number of the case and the control are both N, is*$\frac{8N}{N+2}$.

Proof. We consider Table 1 with a≥0,m≥3,a≤m,a≤N,m≤2N−3, and m−a≤N. The reason for m≥3 and m≤2N−3 is that the 2 × 2 tables above corresponds to a 3 × 2 contingency table with positive margins, which is used for genotype frequency comparisons. The $\chi^{2}$-statistic based on this table can be expressed as a function $\chi^{2}:D\to R_{\geq 0}$, where D={(a,m)∈N∣a≥0,m≥3,a≤m,a≤N,m≤2N−3,m−a≤N}.

Then, we consider the values of (a,m)∈D∩{a≥2,m≥5,m≤2N−3}, which maximize

(1) $$\left|\chi^{2}(a,m)-\chi^{2}(a-2,m-2)\right|.$$

We compute the directional derivative of $\chi^{2}(a,m)$ in direction (−1,−1), and calculate the minimum and maximum value of it. Consequently, (1) takes the maximum value $\frac{8N}{N+2}$ when (a,m)=(N,N). For a detailed proof, see Supplementary Theorem S2 in Supplementary Section S3.1.2. Thus, the sensitivity of $\chi^{2}$-statistics is $\frac{8N}{N+2}$. □

**Table 1.** 2 × 2 contingency table

		Disease status		Total
		0	1	
Allele	0	a	m−a	m
	1	N−a	N−m+a	2N−m
Total		*N*	*N*	2*N*

Similar to the case with a 3 × 2 contingency table, in order to release the *ϵ*-differentially private $\chi^{2}$-statistic, we need to add Laplace noise with scale $\frac{1}{\epsilon }\cdot \frac{8N}{N+2}$ to the true $\chi^{2}$-statistic.

Next, we also describe a method for releasing *ϵ*-differentially private *P*-values. The *P*-values we consider here correspond to the $\chi^{2}$-statistics under the $\chi^{2}$-distribution with 1 degree of freedom.

Theorem 3. *The sensitivity of the P-values obtained from the*$\chi^{2}$*-statistic for allele frequency comparisons based on a*2×2*contingency table, in which the margins are positive and the number of the case and the control are both N, is*$\frac{1}{\sqrt{2\pi }}\int_{0}^{\frac{N}{N-2}}x^{-\frac{1}{2}}\cdot e^{-\frac{x}{2}}\;dx$.

Proof. We consider the same 2×2 contingency table as in Theorem 2. Then the *P*-values can be viewed as a function $p:D\to R_{\geq 0}$, where D={(a,m)∈N∣a≥0,m≥3,a≤m,a≤N,m≤2N−3,m−a≤N}. We consider maximizing

(2) |p(a,m)−p(a−2,m−2)|,

where (a,m)∈D∩{a≥2,m≥5,m≤2N−3}. Then, we can find the value of (2) is maximized when (a,m)=(3,6). For a detailed proof, see Supplementary Theorem S3 in Supplementary Section S3.1.2. Consequently, the sensitivity of *P*-values is

$$\left|p(3,6)-p(1,4)\right|=\frac{1}{\sqrt{2\pi }}\int_{0}^{\frac{N}{N-2}}x^{-\frac{1}{2}}\cdot e^{-\frac{x}{2}}\;dx.$$

□

The value of sensitivity shown in Theorem 3 is approximately equal to 0.682 when *N* is sufficiently large. As with releasing the $\chi^{2}$-statistic, in order to release the *ϵ*-differentially private *P*-value, we can add Laplace noise with scale $\frac{1}{\epsilon }\cdot \frac{1}{\sqrt{2\pi }}\int_{0}^{\frac{N}{N-2}}x^{-\frac{1}{2}}\cdot e^{-\frac{x}{2}}\;dx$. However, as in the case of the χ^2^ test with a 3 × 2 contingency table, the added noise might be much larger than the original *P*-value. Therefore, also in the case of the test with a 2 × 2 table, we consider releasing ${\;\text{log}\;}_{10}$(*P*-values).

Theorem 4. *The sensitivity of*${\;\text{log}\;}_{10}$*(P-values) obtained from the*$\chi^{2}$*-statistic for allele frequency comparisons based on a*2×2*contingency table in which the margins are positive and the number of the case and the control are both N, is less than*2.33.

Proof. The *P*-value corresponding to the $\chi^{2}$-statistic *x* is

$$\frac{1}{\sqrt{2\pi }}\int_{x}^{\infty }x^{-\frac{1}{2}}\cdot e^{-\frac{x}{2}}dx,$$

and we let

$$f(x)={\;\text{log}\;}_{10}\left(\int_{x}^{\infty }x^{-\frac{1}{2}}\cdot e^{-\frac{x}{2}}dx\right).$$

Since the sensitivity of the $\chi^{2}$-statistics is $\frac{8N}{N+2}<8$ [∵ Theorem 2], that of ${\;\text{log}\;}_{10}$(*P*-values) is less than the maximum value of f(x)−f(x+8). We can easily prove that the maximum value is

f(0)−f(8)<2.33.

For a detailed proof, see Supplementary Theorem S4 in Supplementary Section S3.1.2. Consequently, the sensitivity of ${\;\text{log}\;}_{10}$(*P*-values) is less than 2.33. □

Although the exact sensitivity is not shown here, when we add Laplace noise with scale 2.33, the privacy level in differential privacy cannot be reduced. In other words, we can release the *ϵ*-differentially private ${\;\text{log}\;}_{10}$(*P*-values) by this method.

In this section, we have described methods for releasing the $\chi^{2}$-statistics and the *P*-values in the χ^2^ test. It is also important to consider which of these private statistics to employ in practical applications. We will measure their utility in experiments in Section 3 and discuss this point as well.

### 2.2 *ϵ*-Differentially private *P*-values for fisher’s exact test

In statistical tests using a contingency table, the Fisher’s exact test is often used instead of the χ^2^ test when some of the numbers in the cells are small.

#### 2.2.1 Case 1: 2 × 2 contingency table

Similar to Section 2.1, we think about a contingency table for comparing allele frequencies using data from *N* individuals. Firstly, we consider the method of adding noise to the *P*-values themselves and releasing them.

Theorem 5. *The sensitivity of the Fisher’s exact test P-values for allele frequency comparisons based on a*2×2*contingency table, in which the margins are positive and the number of the case and the control are both N, is*$\frac{N(7N-3)}{8(2N-1)(2N-3)}$.

Proof. We consider the same 2×2 contingency table as in Theorem 2. The *P*-value of the Fisher’s exact test obtained from the table is

$$p=\frac{{}_{N}C_{a}\cdot_{N}C_{m-a}}{{}_{2N}C_{m}}.$$

Then we think about the maximum value of

(3) $$\left|\frac{{}_{N}C_{a}\cdot_{N}C_{m-a}}{{}_{2N}C_{m}}-\frac{{}_{N}C_{a-2}\cdot_{N}C_{m-a}}{{}_{2N}C_{m-2}}\right|,$$

where a≥2, m≥5, and m≤2N−3. Considering the cases for the value of *a*, we can see that (3) takes the maximum value when (a,m)=(4,5). For a detailed proof, see Supplementary Theorem S5 in Supplementary Section S3.2.1. Consequently, the sensitivity of the *P*-values is $\frac{N(7N-3)}{8(2N-1)(2N-3)}$. □

When releasing *ϵ*-differentially private *P*-values, we can add Laplace noise with scale $\frac{1}{\epsilon }\cdot \frac{N(7N-3)}{8(2N-1)(2N-3)}$ to the true *P*-values as in Section 2.1.

In the above, we have discussed the releasing method of the *P*-values, but the *P*-value threshold in actual statistical tests is so small that it is well expected to be less than zero when noise is added. Therefore, we also consider releasing ${\;\text{log}\;}_{10}$(*P*-values). In the following, we will show the sensitivity of ${\;\text{log}\;}_{10}$(*P*-values) and explain releasing method of this value as well.

Theorem 6. *The sensitivity of* (${\;\text{log}\;}_{10}$*(P-values) obtained from the Fisher’s exact test for allele frequency comparisons based on a*2×2*contingency table, in which the margins are positive and the number of the case and the control are both N, is*${\;\text{log}\;}_{10}\;\frac{1}{2}(N+1)(N+2))$.

Proof. We consider the same 2×2 contingency table as in Theorem 2. The *P*-value of the Fisher’s exact test obtained from the table is

$$\begin{array}{c} p=\frac{{}_{N}C_{a}\cdot_{N}C_{m-a}}{{}_{2N}C_{m}}\\ =\frac{N!\cdot N!\cdot m!\cdot (2N-m)!}{(2N)!\cdot a!\cdot (m-a)!\cdot (N-a)!\cdot (N-m+a)!}. \end{array}$$

Now we let *f*(*a, m*) be the right side of this equation, then we think about the maximum value of

(4) $$\begin{array}{c} \;\;\;\;\;\;\;\;\left|{\;\text{log}\;}_{10}\left(f(a,m)\right)-{\;\text{log}\;}_{10}\left(f(a-2,m-2)\right)\right|\\ =\left|{\;\text{log}\;}_{10}\left(\frac{f(a,m)}{f(a-2,m-2)}\right)\right|. \end{array}$$

Below, we find the maximum value of

(5) $$\frac{f(a,m)}{f(a-2,m-2)}=\frac{m(m-1)(N-a+1)(N-a+2)}{a(a-1)(2N-m+1)(2N-m+2)}.$$

The smaller the value of *a* and the larger the value of *m*, the larger (5) takes, so we can consider the case of m−a=N. Then

$$\begin{array}{c} (5)=\frac{\left(N+a)(N+a-1\right)}{a(a-1)}\\ =\frac{1}{a(a-1)}N^{2}+\frac{2a-1}{a(a-1)}N+1. \end{array}$$

Therefore, (5) is maximized when (a,m)=(2,N+2), and the maximum value of (4) is ${\;\text{log}\;}_{10}\left(\frac{1}{2}(N+1)(N+2)\right)$. □

This sensitivity highly depends on the value of *N*, and is approximately 3.7, 5.7 and 7.7 when N=100, 1000 and 10 000, respectively. When releasing *ϵ*-differentially private ${\;\text{log}\;}_{10}$(*P*-values), we can use a dataset added Laplace noise with scale $\frac{1}{\epsilon }\cdot {\;\text{log}\;}_{10}\left(\frac{1}{2}(N+1)(N+2)\right)$.

#### 2.2.2 Case 2: 3 × 2 contingency table

Here, we consider the case with a 3 × 2 contingency table for comparing genotype frequencies. In the following, we will present a method for releasing ${\;\text{log}\;}_{10}$(*P*-values) obtained from the test as in the case with a 2 × 2 contingency table.

Theorem 7. *The sensitivity of*${\;\text{log}\;}_{10}$*(P-values) obtained from the Fisher’s exact test for genotype frequency comparisons based on a*3×2*contingency table, in which the margins are positive and the number of the case and the control are both*N/2*, is*${\;\text{log}\;}_{10}\left(\frac{N}{2}+1\right)$*.’*

Proof. We can prove this easily in the similar way to Theorem 6. For a detailed proof, see Supplementary Theorem S7 in Supplementary Section S3.2.2. □

Similar to the case with a 2 × 2 contingency table, the sensitivity highly depends on the value of *N*, and approximately 1.7, 2.7 and 3.7 when N=100, 1000 and 10 000, respectively. In order to release *ϵ*-differentially private ${\;\text{log}\;}_{10}$(*P*-values), we have to add Laplace noise with scale $\frac{1}{\epsilon }\cdot {\;\text{log}\;}_{10}\left(\frac{N}{2}+1\right)$.

### 2.3 *ϵ*-Differentially private statistics for Cochran–Armitage’s trend test

Besides the χ^2^ test and the Fisher’s exact test, statistical tests using 3 × 2 contingency tables include the Cochran–Armitage’s trend test used to test whether there is a trend between a variable with two categories such as disease status and an ordinal variable with three categories like genotypes. In this section, we describe methods for releasing the $\chi^{2}$-statistics and *P*-values obtained by the Cochran–Armitage’s trend test while maintaining privacy.

Theorem 8. *The sensitivity of the*$\chi^{2}$*-statistics of the Cochran–Armitage’s trend test based on a*3×2*contingency table, in which the margins are positive and the number of the case and the control are both*N/2*, is*$\frac{16N(N^{2}+6N+4)}{\left(N+18)(N^{2}+8N-4\right)}$.

Proof. We consider Table 2 with a≥0,b≥0,m>0,n>0,a≤m,b≤n,a+b≤N/2,m+n<N, and m+n−a−b≤N/2. The $\chi^{2}$-statistic of the Cochran–Armitage’s trend test obtained from the table is

$$T=\frac{N{\left(2m+n-2(2a+b)\right)}^{2}}{4Nm+Nn-{\left(2m+n\right)}^{2}}.$$

This statistic can be expressed as a function $\chi^{2}:D\to R_{\geq 0}$, where D={(a,b,m,n)∈N∣a≥0,b≥0,m>0,n>0,a≤m,b≤n,a+b≤N/2,m+n<N,m+n−a−b≤N/2}. Now we consider maximizing

(6) $$\left|\chi^{2}(a-1,b,m-1,n)-\chi^{2}(a,b,m,n)\right|$$

where (a,b,m,n)∈D∩{a≥1,m≥2}. Then, we can prove that the maximum value of (6) is $\frac{16N(N^{2}+6N+4)}{\left(N^{2}+8N-4)(N+18\right)}$ when (a,b,m,n)=(1,0,2,N/2−1), and this value is the sensitivity of $\chi^{2}$-statistics. For a detailed proof, see Supplementary Theorem S8 in Supplementary Section S3.3. □

**Table 2.** 3 × 2 contingency table

		Disease status		Total
		0	1	
Genotype	0	a	m−a	*m*
	1	*b*	n−b	*n*
	2	N/2−a−b	N/2−m−n+a+b	N−m−n
Total		N/2	N/2	*N*

When we add Laplace noise with scale $\frac{1}{\epsilon }\cdot \frac{16N(N^{2}+6N+4)}{\left(N^{2}+8N-4)(N+18\right)}$ to the true $\chi^{2}$-statistic, we can release the *ϵ*-differentially private $\chi^{2}$-statistic.

In the following, we describe a method for releasing the *P*-values in the Cochran–Armitage’s trend test as their base 10 logarithms.

Theorem 9. *The sensitivity of*${\;\text{log}\;}_{10}$*(P-values) obtained from the*$\chi^{2}$*-statistic of the Cochran–Armitage’s trend test based on a*3×2*contingency table, in which the margins are positive and the number of the case and the control are both*N/2*, is*${\;\text{log}\;}_{10}(e)\cdot \frac{8N(N^{2}+6N+4)}{\left(N+18)(N^{2}+8N-4\right)}$.

Proof. We can prove this easily in the similar way as Theorem 1. For a detailed proof, see Supplementary Theorem S9 in Supplementary Section S3.3. □

As in the case of the χ^2^ test and the Fisher’s exact test, we can add Laplace noise with scale $\frac{1}{\epsilon }\cdot {\;\text{log}\;}_{10}(e)\cdot \frac{8N(N^{2}+6N+4)}{\left(N^{2}+8N-4)(N+18\right)}$ when releasing *ϵ*-differentially private ${\;\text{log}\;}_{10}$(*P*-values). Incidentally, the value of sensitivity shown in Theorem 9 is around 3.47 when *N* is large enough.

In this paper, we considered the case where the number of the case is equal to the number of the control. In Supplementary Section S3.4, we discussed a little about the value of sensitivity when they are different. However, we believe that further research is required for more rigorous theoretical guarantees.

## 3 Experiments and discussion

We measured the utility of the private statistics by calculating the KL divergence (Kullback and Leibler, 1951) between the original statistics and the noise-added statistics by our experiments. In this study, we adopted KL divergence instead of L1 or L2 norm in order to evaluate the difference between two distributions of these statistics (Kosheleva and Kreinovich, 2017). The definition of KL divergence that we used in our experiments is as follows:

Definition 3. *For discrete probability distributions p and q defined on the same probability space X, the KL divergence is defined by*

$$D_{\text{KL}}(p||q)=\sum_{x\in X}p(x)\;\text{log}\;\frac{p(x)}{q(x)}.$$

When evaluating the methods for $\chi^{2}$-statistics, we considered $\chi^{2}$-statistics from 10 to 100 in increments of 10. For each of these values, we applied the method presented in Section 2 to 10 000 datasets and calculated the KL divergence between the statistics from the resulting datasets and those from the original datasets. When evaluating the methods for *P*-values, we considered *P*-values such that the value of $-{\;\text{log}\;}_{10}$(*P*-value) ranged from 0 to 20, in two increments. This is because the threshold of the *P*-values is often set to $5\times 10^{-8}$ in general GWAS. The same method was used to evaluate the utility of the private base 10 logarithms of the *P*-values. Based on the number of participants in a typical GWAS, we considered the cases where the number of individuals in the simulation data was *N* = 1000, 10 000, 50 000 and 100 000.

The value of *ϵ* in differential privacy was considered to be in the range from 0.1 to 10. The reason for this is that the range of *ϵ* in studies where differential privacy was applied is mostly from 0.01 to 10 (Hsu *et al.*, 2014). When *ϵ* is less than 0.1, the added noise is very large compared to the original statistics, so we set the minimum value of *ϵ* to 0.1.

Then, we conducted experiments to determine thresholds of $\chi^{2}$-statistics and those of *P*-values to be used when the noise-added statistics are applied practically. In the statistical tests considered in this paper, it is assumed that the $\chi^{2}$-statistics roughly have a $\chi^{2}$-distribution with degrees of freedom for each test method and that the corresponding *P*-values follow an approximately uniform distribution. Therefore, we generated the datasets for the simulation study based on the distribution that each statistics is expected to follow. Specifically, the statistics for $10^{9}$ individuals were generated as random numbers so that they would follow a $\chi^{2}$-distribution for the $\chi^{2}$-statistics and a normal distribution for the *P*-values. In these datasets, data above the original threshold are the data to be tested as statistically significant. Here, the original *P*-value threshold is $5\times 10^{-8}$, and the corresponding $\chi^{2}$-statistic is 29.7 for the test using a 2×2 contingency table and 33.6 for the test using a 3×2 table. We added noise to these datasets by using the methods shown in Section 3 and measured the change in the values of precision, recall, and *f*-measure as we changed the thresholds to find an appropriate threshold for high accuracy. The detailed calculation of the values of precision, recall and *f*-measure is shown in Supplementary Section S1.5.

The value range of the thresholds considered in this experiment was set according to the original thresholds. The total number of individuals in the dataset was set to *N* = 100 000 for the χ^2^ test and the Cochran–Armitage’s trend test. While for the Fisher’s exact test, we considered the cases when N=100 and 1000, since the added noise depends heavily on the value of *N* and the noise is too large to be applied if *N* ≥ 10 000.

In Supplementary Section S4.4, we show the results of applying our method to a real dataset. The dataset we used is UKB MDD data by Coleman *et al.* (2020) provided in LD Hub (Zheng *et al.*, 2017).

### 3.1 *ϵ*-Differentially private statistics for χ^2^ test

#### 3.1.1 Case 1: 3 × 2 contingency table

**Fig. 1.**
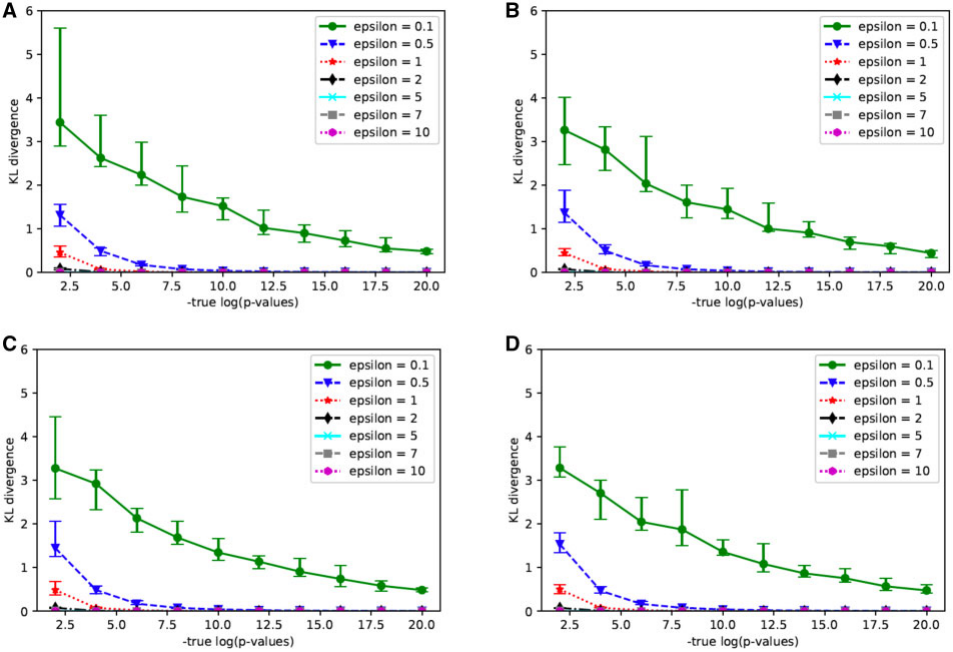
KL divergence between the original and the private $-{\;\text{log}\;}_{10}$(*P*-values) in χ^2^ test with a 3×2 contingency table when (**A**) N=1000, (**B**) N=10 000, (**C**) N=50 000 and (**D**) N=100 000

We considered the method for releasing *P*-values. In previous research, Fienberg *et al.* (2011) proposed a method to release the *P*-values themselves, but it is not very practical due to the excessive amount of noise. In the following, we assessed the utility of our method to release private ${\;\text{log}\;}_{10}$(*P*-values). Firstly, we obtained the KL divergence between the original and the private statistics. Here, we generated datasets with noise based on Theorem 1. Figure 1 shows the KL divergence obtained in this experiment.

Since the added noise increases with a smaller value of *ϵ* as shown in Section 2, the KL divergence is also highly dependent on the value of *ϵ* in Figure 1. On the other hand, for the total number of individuals *N* in a dataset, adding noise is almost the same if *N* is large enough. In fact, there is little change in the sketch in the four graphs above. One common feature of these graphs is that the smaller $-{\;\text{log}\;}_{10}$(*P*-values), the larger the KL divergence. This may be due to the fact that noise-added statistics which have become smaller than zero must be rounded to over zero. However, from Figure 1, it is demonstrated that it might be practically possible to release *ϵ*-differentially private ${\;\text{log}\;}_{10}$(*P*-values) of the *P*-values if *ϵ* is greater than or equal to 2.

**Fig. 2.**
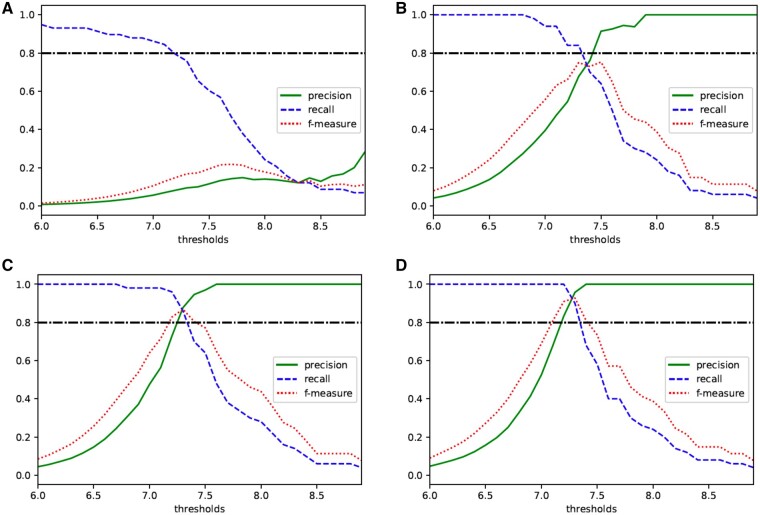
Relationship between thresholds of private $-{\;\text{log}\;}_{10}$(*P*-values) and precision, recall and *f*-measure in χ^2^ test with a 3×2 contingency table when (**A**) ϵ=2, (**B**) ϵ=5, (**C**) ϵ=7 and (**D**) ϵ=10

Next, we considered the appropriate thresholds when the *ϵ* is 2, 5, 7 and 10. We note that the general threshold of *P*-values in GWAS is $5\times 10^{-8}$. In this case, the $-{\;\text{log}\;}_{10}$(*P*-values) threshold is almost 7.3. Therefore, we set the private thresholds from 6.0 to 9.0 in increments of 0.1. Then, we calculated precision, recall and *f*-measure for each threshold and the results are shown in Figure 2.

When ϵ=2, the *f*-measure is maximized by the threshold to 7.7. However, the value is too small to use in practical, suggesting that the value of *ϵ* has to be larger than 2. In the other three cases, the *f*-measures are maximized when the threshold is set to 7.3, and the maximum values are over 0.8. Therefore, these figures imply that setting *ϵ* to 5, 7 and 10 in practical use is not a problem. The larger the value of *ϵ*, the lower the privacy level achieved, so when using our method for actual tests, it will be appropriate to set *ϵ* to 5 or 7, and the threshold to 7.3. The above discussion indicates that the *P*-values in the χ^2^ test with a 3 × 2 table can be released privately by taking their base 10 logarithms.

#### 3.1.2 Case 2: 2 × 2 contingency table

In this section, we evaluated our methods for releasing the statistics in the χ^2^ test using a 2 × 2 contingency table.

Firstly, in order to assess the utility of the private $\chi^{2}$-statistics, we obtained the KL divergence between the original and the private $\chi^{2}$-statistics. Supplementary Figure S1 shows that our method to release *ϵ*-differentially private $\chi^{2}$-statistics might be useful if *ϵ* is greater than or equal to 5. Therefore, we consider the thresholds when the *ϵ* is 5, 7 and 10 in the following.

We note that the degree of freedom for the χ^2^ test using a 2 × 2 contingency table is 1 and the general threshold of *P*-values in GWAS is $5\times 10^{-8}$. In this case, the $\chi^{2}$-statistic corresponding to the *P*-value threshold is approximately 29.7. Therefore, we varied thresholds from 25 to 34.9 in increments 0.1. As in Case 1, we examined the appropriate thresholds for the private $\chi^{2}$-statistics by calculating precision, recall and *f*-measure.

**Fig. 3.**
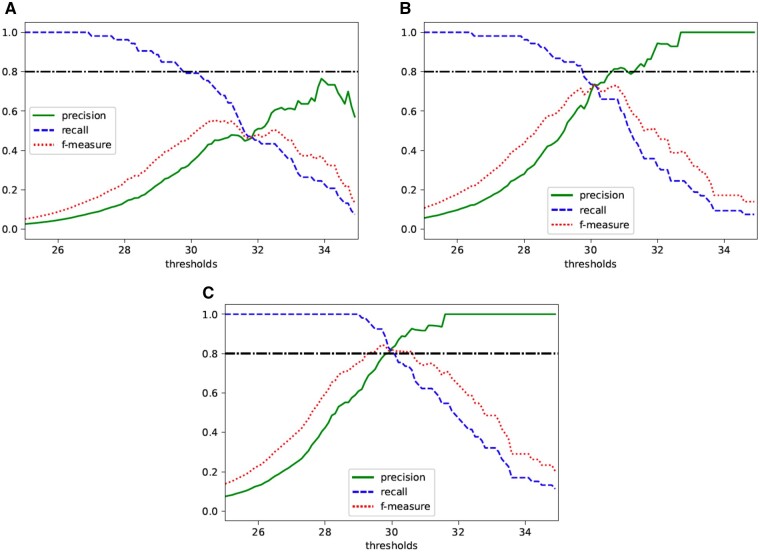
Relationship between thresholds of private $\chi^{2}$-statistics and precision, recall and *f*-measure in χ^2^ test with a 2×2 contingency table when (**A**) ϵ=5, (**B**) ϵ=7 and (**C**) ϵ=10

Figure 3 shows the relationship between thresholds and these values. When ϵ=5, the *f*-measure is maximized by setting the threshold to 30.7. However, the precision is less than 0.6 at this time, suggesting a lack of practicality compared to the case of ϵ=7. When ϵ=7, the *f*-measure is at its maximum when the threshold is 30.1, and the precision is about 0.8 at this time. Therefore, it is implied that it is acceptable to set *ϵ* to 7 in practical use. If higher precision is desired, a threshold of 33 seems to be a good choice. When ϵ=10, the threshold that maximizes *f*-measure is 29.7, which is almost the same as the original threshold. Even when the threshold is set to around 30.5, the precision is greater than 0.9 and *f*-measure is also greater than when ϵ=7. Thus, we can conclude that our method when ϵ=10 is quite useful. Hence, when using *ϵ*-differentially private $\chi^{2}$-statistics for actual tests, it might be appropriate for high accuracy to set the value of *ϵ* to 7 or 10, and the threshold to 30.1 or 30.5 in each case.

In the supplement, we also evaluated the utility of private *P*-values and $-{\;\text{log}\;}_{10}$(*P*-values).

### 3.2 *ϵ*-Differentially private *P*-values for fisher’s exact test

In this section, we discuss the utility of our method for releasing private *P*-values in the Fisher’s exact test. In the case with a 2 × 2 contingency table, the method for releasing *P*-values and that for releasing ${\;\text{log}\;}_{10}$(*P*-values) were evaluated. And in the case with a 3 × 2 contingency table, the method for releasing ${\;\text{log}\;}_{10}$(*P*-values) was evaluated.

#### 3.2.1 Case 1: 2 × 2 contingency table

As in the case of χ^2^ test, we assessed the practicality of our methods for releasing the private *P*-values and $-{\;\text{log}\;}_{10}$(*P*-values). The details of these experiments are shown in the supplement, and the results show that it might be possible to maintain both privacy and utility by considering the ${\;\text{log}\;}_{10}$(*P*-values) when using and releasing private statistics in the Fisher’s exact test. However, our method can be applied only when *N* is small and *ϵ* is reasonably large. Therefore, in the future, it is necessary to develop the test methods specifically for the case of *N* is large and to study the risk of privacy violation by increasing the value of *ϵ*.

#### 3.2.2 Case 2: 3 × 2 contingency table

**Fig. 4.**
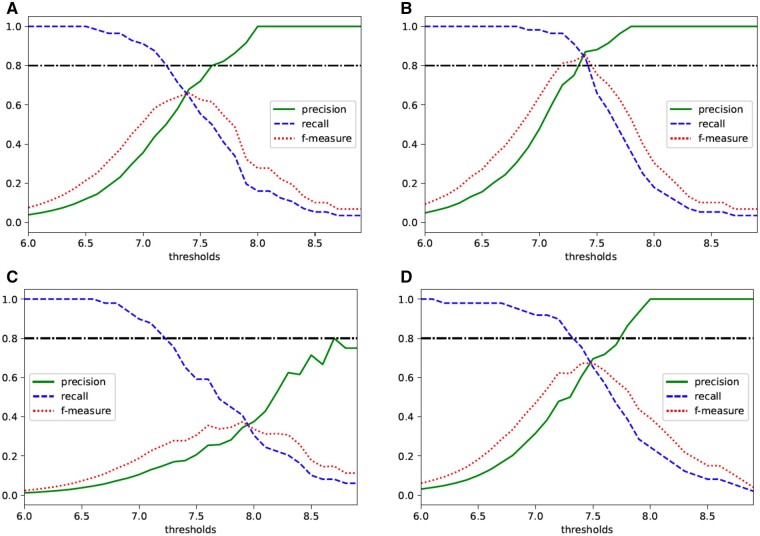
Relationship between thresholds of private $-{\;\text{log}\;}_{10}$(*P*-values) and precision, recall and *f*-measure in the Fisher’s exact test with a 3×2 contingency table when (**A**) N=100, ϵ=7, (**B**) N=100, ϵ=10, (**C**) N=1000, ϵ=7 and (**D**) N=1000, ϵ=10

As in the case with a 2 × 2 contingency table, we evaluated the method for releasing ${\;\text{log}\;}_{10}$(*P*-values). Firstly, we calculated the KL divergence from the original and the private $-{\;\text{log}\;}_{10}$(*P*-values) in Supplementary Section S4.2.2. Then, we considered the thresholds by calculating precision, recall and *f*-measure similar to Section 3.2.1. Figure 4 shows the results. When N=100, the maximum value of *f*-measure is sufficiently large in both cases where ϵ=7 and 10. Therefore, our releasing method seems to be practical. The appropriate threshold for practical use would be the point where the *f*-measure is maximized, i.e., 7.4. When N=1000, our method could be useful if we set the value of *ϵ* to 10. In this case, the *f*-measure takes the maximum value when the threshold is 7.5, and the precision value at this point is around 0.7, so the threshold should be set to 7.5.

The above results suggest that our method for releasing ${\;\text{log}\;}_{10}$(*P*-values) in Fisher’s exact test with a 3 × 2 table is more practical than that with a 2 × 2 table. However, even in this case, the added noise becomes larger as the total number of individuals *N* becomes larger, and it seems that our method cannot be used for very large datasets.

### 3.3 *ϵ*-Differentially private statistics for Cochran–Armitage’s trend test

**Fig. 5.**
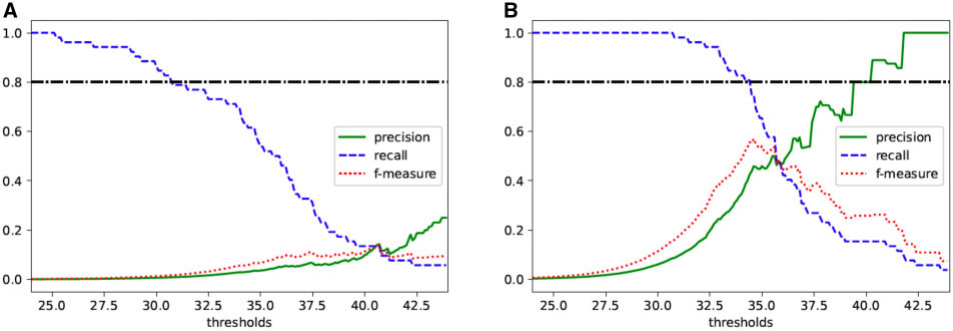
Relationship between thresholds of private $\chi^{2}$-statistics and precision, recall and *f*-measure in the Cochran–Armitage’s trend test when (**A**) ϵ=7 and (**B**) ϵ=10

Similar to the case of the χ^2^ test, we calculated the KL divergence for the $\chi^{2}$-statistics and the results are shown in Supplementary Figure S5. From this figure, we can assume that the value of *ϵ* acceptable for practical use is around 7 and 10. Therefore, we considered the thresholds when *ϵ* is set to 7 and 10. Since the degree of freedom of the Cochran–Armitage’s trend test with a 3 × 2 contingency table is 2, the $\chi^{2}$-statistic corresponding to the original *P*-value threshold of $5\times 10^{-8}$ is approximately 33.6. Thus, in this experiment, we set the threshold for using the private $\chi^{2}$-statistics from 24 to 44 in increments of 0.1. For each of these thresholds, we calculated precision, recall and *f*-measure as in Sections 3.1 and 3.2, and plotted them in Figure 5. When ϵ=7, *f*-measure is maximized when threshold is 40.7. At this time, precision is less than 0.2, and it is not very practical to set epsilon to 7. When ϵ=10, *f*-measure takes the maximum value at threshold of 34.6. However, precision at this point is less than 0.6. If the value of precision is prioritized, it is indicated that the threshold should be set around 37.5 to 38.0.

Next, we evaluated the method for releasing *P*-values in the Cochran–Armitage’s trend test. We considered releasing ${\;\text{log}\;}_{10}$(*P*-values) and adding noise based on Theorem 9. Similar to the case of the Fisher’s exact test, we calculated the KL divergence between the original and the private $-{\;\text{log}\;}_{10}$(*P*-values). Supplementary Figure S6 shows these results. From the figure, we will find the appropriate thresholds for the cases of ϵ=7 and 10.

**Fig. 6.**
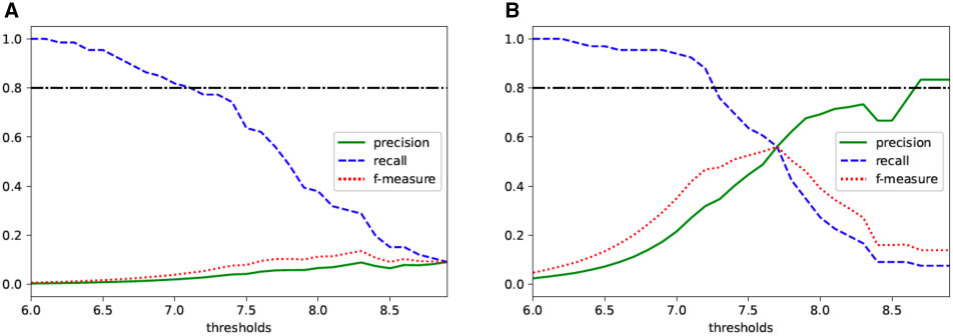
Relationship between thresholds of private $-{\;\text{log}\;}_{10}$(*P*-values) and precision, recall and *f*-measure in the Cochran–Armitage’s trend test when (**A**) ϵ=7 and (**B**) ϵ=10

Figure 6 shows the precision, recall and f-measure when the thresholds are varied as in Sections 3.1 and 3.2. When ϵ=7. Both precision and *f*-measure are very small, implying that this epsilon value is not practical at all. When ϵ=10, *f*-measure takes the maximum value when the threshold is set to 7.7. Since the precision at this time is around 0.6, it might be better to set it around 8.0 in practical terms.

These results indicate that our methods can be used to some extent for both the $\chi^{2}$-statistics and the *P*-values of the Cochran–Armitage’s trend test. However, it was implied that the releasing methods are not as practical as the methods in the χ^2^ test. In fact, the noise we added in this section is about from 1.5 to 2 times larger than that in Section 3.1. One possible reason for this is that in the case of the $\chi^{2}$ test, a change in one individual’s data is equivalent to a change in one allele, but in the Cochran–Armitage’s trend test, it is necessary to consider that two alleles may change. Therefore, when considering the sensitivity of statistics in the tests on genomic data, further discussion will be required to determine whether we must consider a single individual or just one allele.

## 4 Conclusion

In this paper, we have shown how to conduct statistical tests with contingency tables in GWAS while preserving the privacy of individuals. In addition to the privacy-preserving statistical tests mentioned in previous studies (e.g. Fienberg *et al.*, 2011), we have covered all statistical testing methods used in GWAS. For private *P*-values, we have solved the problem of low utility due to the fact that the added noise is much larger than the original *P*-value threshold by considering their base 10 logarithms. Furthermore, we have also shown the appropriate thresholds with high accuracy for private statistics obtained by applying our methods. From our experimental results, it has been indicated that our methods may be practical for the χ^2^ test and the Cochran–Armitage’s trend test. For the Fisher’s exact test, our results suggest that our methods could be applicable when the total number of individuals in the dataset is small.

However, the utility of the methods for the Fisher’s exact test and the Cochran–Armitage’s test is lower than that of the methods for the χ^2^ test. This result raises the question of whether to consider a single individual or a single allele when calculating sensitivity in a genomic dataset. In other words, there needs to be further study on what is an acceptable level of privacy when the neighboring of datasets is defined by information about a single allele. If we can only focus on a single allele, the amount of noise will be much less than our methods. Moreover, the dependencies between genomes are not taken into account in this paper. In fact, the larger the number of SNPs to be released, the smaller the epsilon value to be set because of the dependencies among SNPs. More specifically, the concept proposed by Zhao *et al.* (2017) or the definition by Almadhoun *et al.* (2020) could be used for genomic datasets. Then, it is necessary to develop our methods to take dependencies into account and conduct further research on their application to more real datasets. In addition, further development of releasing methods for other statistics such as *P*-values in family-based control studies is also desired.

For further research on our methods, it might be worthwhile to focus only on data around the threshold in order not to consider the value range of statistics. For data that are far from the original thresholds, it may be possible to use random values within a certain range because the values of recall or precision are high almost regardless of the amount of noise.

## Supplementary Material

vbab004_Supplementary_Data

## References

[vbab004-B1] Abadi M. et al (2016) Deep learning with differential privacy. CCS '16: Proceedings of the 2016 ACM SIGSAC Conference on Computer and Communications Security, 308–318.

[vbab004-B2] Almadhoun N. et al (2020) Differential privacy under dependent tuples-the case of genomic privacy. Bioinformatics, 36, 1696–1703. 31702787 10.1093/bioinformatics/btz837

[vbab004-B3] Armitage P. (1955) Tests for linear trends in proportions and frequencies. Biometrics, 11, 375–386.

[vbab004-B4] Chen C.-W. , Yang H.-C. (2019) OPATs: omnibus p-value association tests. Brief. Bioinform., 20, 1–14. 28981573 10.1093/bib/bbx068 PMC6357551

[vbab004-B5] Coleman J.R.I. et al (2020) The genetics of the mood disorder spectrum: genome-wide association analyses of more than 185,000 cases and 439,000 controls. Biol. Psychiatry, 88, 169–184. 31926635 10.1016/j.biopsych.2019.10.015 PMC8136147

[vbab004-B6] Dickhaus T. et al (2012) How to analyze many contingency tables simultaneously in genetic association studies. Stat. Appl. Genet. Mol. Biol., 11, Article 12. doi:10.1515/1544-6115.1776. 10.1515/1544-6115.1776 22850061

[vbab004-B7] Dwork C. (2006). Differential privacy. In: Bugliesi M. *et al.* (eds.) Automata, Languages and Programming, ICALP 2006, Lecture Notes in Computer Science, vol 4052. Springer, Berlin, Heidelberg, p. 4052.

[vbab004-B8] Dwork C. et al (2006). Calibrating noise to sensitivity in private data analysis. In: Halevi S., Rabin T. (eds.) Theory of Cryptography, TCC 2006, Lecture Notes in Computer Science, vol 3876. Springer, Berlin, Heidelberg, p. 3876.

[vbab004-B9] Fienberg S.E. et al (2011). Privacy preserving GWAS data sharing. In: *IEEE 11th International Conference on Data Mining Workshops.* pp. 628–635.

[vbab004-B10] Fisher R.A. (1935). The Design of Experiments. Oliver and Boyd. Edinburgh and London.

[vbab004-B11] Homer N. et al (2008) Resolving individuals contributing trace amounts of DNA to highly complex mixtures using high-density SNP genotyping microarrays. PLoS Genet., 4, e1000167. 18769715 10.1371/journal.pgen.1000167 PMC2516199

[vbab004-B12] Hsu J. et al (2014). Differential privacy: an economic method for choosing epsilon. In: *2014 IEEE Computer Security Foundations Symposium.* pp. 398–410.

[vbab004-B13] Kosheleva O. , Kreinovich V. (2017) Why deep learning methods use KL divergence instead of least squares: a possible pedagogical explanation. Math. Struct. Model., 46, 102–106.

[vbab004-B14] Kullback S. , Leibler R.A. (1951) On information and sufficiency. Ann. Math. Statist., 22, 79–86.

[vbab004-B15] Matthews A.G. et al (2008) Collapsing SNP genotypes in case-control genome-wide association studies increases the type I error rate and power. Stat. Appl. Genet. Mol. Biol., 7, Article23. doi:10.2202/1544-6115.1325. 10.2202/1544-6115.1325 PMC2789285 18673292

[vbab004-B16] Raisaro J.L. et al (2019) MedCo: enabling secure and privacy-preserving exploration of distributed clinical and genomic data. IEEE/ACM Trans. Comput. Biol. Bioinform., 16, 1328–1341. 30010584 10.1109/TCBB.2018.2854776

[vbab004-B17] Spielman R.S. et al (1993) Transmission test for linkage disequilibrium: the insulin gene region and insulin-dependent diabetes mellitus (IDDM). Am. J. Hum. Genet, 52, 506–516. 8447318 PMC1682161

[vbab004-B18] Wang R. et al (2009) Learning your identity and disease from research papers: information leaks in genome wide association study. CCS '09: Proceedings of the 16th ACM Conference on Computer and Communications Security, 534–544.

[vbab004-B19] Weber G.M. et al (2009) The Shared Health Research Information Network (SHRINE): a prototype federated query tool for clinical data repositories. J. Am. Med. Inform. Assoc., 16, 624–630. 19567788 10.1197/jamia.M3191 PMC2744712

[vbab004-B20] Yates F. (1934) Contingency tables involving small numbers and the $\chi^{2}$ test. Suppl. J. R. Stat. Soc., 1, 217–235.

[vbab004-B21] Zaykin D.V. , Kozbur D.O. (2010) P-value based analysis for shared controls design in genome-wide association studies. Genet. Epidemiol., 34, 725–738. 20976797 10.1002/gepi.20536 PMC3190645

[vbab004-B22] Zeng P. et al (2015) Statistical analysis for genome-wide association study. J. Biomed. Res., 29, 285–297. 26243515 10.7555/JBR.29.20140007 PMC4547377

[vbab004-B23] Zerhouni E.A. , Nabel E.G. (2008) Protecting aggregate genomic data. Science, 322, 44. 10.1126/science.322.5898.44b 18772394

[vbab004-B24] Zhao J. et al (2017). Dependent differential privacy for correlated data. In: *2017 IEEE Globecom Workshops (GC Wkshps)*. pp. 1–7.

[vbab004-B25] Zheng J. et al (2017) LD Hub: a centralized database and web interface to perform LD score regression that maximizes the potential of summary level GWAS data for SNP heritability and genetic correlation analysis. Bioinformatics, 33, 272–279. 27663502 10.1093/bioinformatics/btw613 PMC5542030

