# Supplementary material for: More practical differentially private publication of key statistics in GWAS
Source: Bioinform Adv. 2021 May 18;1(1):vbab004. doi: 10.1093/bioadv/vbab004 (PMC9710635; doi:10.1093/bioadv/vbab004)
Supplement: vbab004_Supplementary_Data [file vbab004_supplementary_data.pdf]

In Section S1, we describe basic assumptions and preliminary definitions in our study. We overview the related work in Section S2. In Section S3, we give detailed proofs for the theoretical guarantees of our methods. In Section S4, we provide supplemental results and discussion in our experiments.

S1 Preliminaries

This section descries basic assumptions and definitions for the statistical tests, differential privacy, and analyses of the experimental results.

S1.1 Chi-squared Test

In this study, we consider the chi-squared test with a 3 × 2 contingency table and that with a 2 × 2 contingency table. For a $K \times 2$ table t with counts $t_{i,j}$ and row sums s_i, the χ^2-statistic is

$$\chi^2(t) = \sum_{i=1}^{K} \frac{(t_{i,1} - t_{i,2})^2}{s_i}.$$

Under the null χ^2-distribution, the p-value corresponding to a value x of the χ^2-statistic with a 3 × 2 table is

$$p = \mathrm{e}^{-\frac{x}{2}},$$

and that with a 2 × 2 table is

$$p = \frac{1}{\sqrt{2\pi}} \int_x^\infty x^{-\frac{1}{2}} \cdot \mathrm{e}^{-\frac{x}{2}}\, \mathrm{d}x.$$

S1.2 Fisher's Exact Test

The Fisher's exact test (Fisher, 1935) is a statistical significance test that is often used when there are small numbers in some cells of a contingency table. Whereas the chi-squared test calculates the p-value from the χ^2-statistic, this test gets the p-value directly from the table data.

Table S1. Allele counts distribution for the case-control studies.

		Disease Status 0	Disease Status 1	Total
Allele	0	a	$m-a$	m
	1	$N-a$	$N-m+a$	$2N-m$
Total		N	N	$2N$

Table S2. Genotype counts distribution for the case-control studies.

		Disease Status 0	Disease Status 1	Total
Genotype	0	a	$m-a$	m
	1	b	$n-b$	n
	2	$N/2-a-b$	$N/2-m-n+a+b$	$N-m-n$
Total		$N/2$	$N/2$	N

We consider the case of an allele frequency comparison using a 2 × 2 contingency table shown in Table S1 and the case of a genotype frequency comparison using a 3 × 2 contingency table shown in Table S2 where the total number of individuals is N. Here, we note that each individual has two alleles, and the total number of alleles in Table S1 is $2N$. For the case with Table S1, the p-value obtained from the Fisher's exact test is

$$\begin{aligned} p &= \frac{{}_m\mathrm{C}_a \cdot {}_{2N-m}\mathrm{C}_{N-a}}{{}_{2N}\mathrm{C}_N} = \frac{{}_N\mathrm{C}_a \cdot {}_N\mathrm{C}_{m-a}}{{}_{2N}\mathrm{C}_m} \\ &= \frac{N! \cdot N! \cdot m! \cdot (2N-m)!}{(2N)! \cdot a! \cdot (m-a)! \cdot (N-a)! \cdot (N-m+a)!}, \end{aligned}$$

and for the case with Table S2,

$$p = \frac{{}_m\mathrm{C}_a \cdot {}_n\mathrm{C}_b \cdot {}_{N-m-n}\mathrm{C}_{N/2-a-b}}{{}_N\mathrm{C}_{N/2}}.$$

S1.3 Cochran-Armitage's Trend Test

The Cochran-Armitage's trend test (Armitage, 1955) is commonly used to determine if there is a trend among binomial proportions in studies where the underlying genetic model is unknown (Ghodsi *et al.*, 2016). Here we consider the test for a 3 × 2 contingency table, and we assume that the genotype counts for the case and those for the control follow independent multinomial distributions with parameters (p_0, p_1, p_2), (p'_0, p'_1, p'_2), respectively, where the parameters are the genotype probabilities in the case and the control. When we take the assumption of no trend to be the null hypothesis, $H_0 : p_i = p'_i$ for $i = 0, 1, 2$. In order to test whether the major and minor alleles are codominant, the weights used in the test are set as $(t_0, t_1, t_2) = (0, 1, 2)$.

Similar to the case of the Fisher's exact test, we consider the 3 × 2 contingency table shown in Table S2. The χ^2-statistic in the Cochran-Armitage's trend test for the data in Table S2 is given by

$$T = \frac{N(2m+n-2(2a+b))^2}{4Nm+Nn-(2m+n)^2}.$$

This test based on a 3 × 2 table has 2 degrees of freedom, and the p-value corresponding to a value x of the χ^2-statistic under the null χ^2-distribution is

$$p = \mathrm{e}^{-\frac{x}{2}}.$$

S1.4 Differential Privacy

Differential privacy (Dwork, 2006) is a theoretical framework developed in the field of cryptography to protect the validity of data while preventing the leakage of personal information contained in a dataset. The idea of differential privacy is based on that it should be sufficiently difficult to find differences between statistics derived from the original dataset and that calculated from a dataset where only one individual is different from the original dataset. The value of the parameter $\epsilon > 0$ determines the level of privacy, with ϵ closer to zero indicating more privacy protection. On the contrary, with increasing the value of ϵ, the degree of privacy assurance becomes less, but the accuracy and utility of the private data increased. Therefore, it is very important to consider the trade-off between privacy and utility and to set an appropriate ϵ. The following is the definition of ϵ-differentially privacy.

Definition S1 . *A randomized mechanism M is ϵ-differentially private if, for all datasets D and D' which differ in only one individual and any S $\subset$range(M),*

$$\Pr[M(D) \in S] \leq e^{\epsilon} \cdot \Pr[M(D') \in S].$$

To satisfy the definition of ϵ-differential privacy, the *sensitivity* of a function is often considered. The definition of the *sensitivity* is as follows.

Definition S2 . *Let $\mathcal{D}^N$ be the collection of all datasets with N individuals, the sensitivity of a function $f : \mathcal{D}^N \to \mathbb{R}^d$ is*

$$\Delta f = \max_{D,D'} \|f(D) - f(D')\|_1,$$

where $D, D' \in \mathcal{D}^N$ differ in a single individual.

For a statistic $f(D)$ obtained from the original dataset D, releasing $f(D) + b$ satisfies ϵ-differential privacy when b is random noise derived from a Laplace distribution with mean 0 and scale $\frac{\Delta f}{\epsilon}$ (Dwork *et al.*, 2006). This releasing method is often called as the Laplace mechanism.

S1.5 Analyses of the experimental results

In our study, we use KL divergence (Kullback and Leibler, 1951) to measure the difference between two distributions of the original statistic and its noise-added version. In this study, we adopted KL divergence instead of L1 or L2 norm because the added noise is not normally distributed (Kosheleva and Kreinovich, 2017).

Definition S3 . *For discrete probability distributions p and q defined on the same probability space X, the KL divergence is defined by*

$$D_{\mathrm{KL}}(p\|q) = \sum_{x \in X} p(x) \log \frac{p(x)}{q(x)}.$$

In addition, we calculated the values of precision, recall, and f-measure to find appropriate private thresholds. When finding these values, we considered the following table. In the table, Th corresponds to the original threshold of statistics.

		original statistics		Total
		$> Th$	$\leq Th$	
private statistics	$>$ threshold	tp	fp	$tp + fp$
	$\leq$ threshold	fn	tn	$fn + tn$
Total		$tp + fn$	$fp + tn$	10^9

$$\begin{aligned} Precision &= \frac{tp}{tp + fp}, \\ Recall &= \frac{tp}{tp + fn}, \\ F{-}measure &= 2 \times \frac{Precision \times Recall}{Precision + Recall}. \end{aligned}$$

S2 Related Work

In this section, we overview the related work on GWAS data handling methods and differential privacy techniques for dealing genomic data.

S2.1 GWAS

A problem in the application of differential privacy techniques to GWAS data is that they are limited in scope and flexibility of adaptation. For example, researchers who want to analyze GWAS data may be required to know the location of disease-related SNPs and characteristics of the data in advance. In such a situation, Johnson and Shmatikov, 2015 proposed a privacy-preserving query framework to allow researchers to explore data regardless of their background knowledge. Their framework allows analysts to learn information about SNPs associated with diseases and correlations in the genome in a privacy-preserving manner. However, one problem with this study is that no assurances were made as to how accurate the output obtained by the query is, making its usefulness in the medical research phase questionable.

As we can see from the above study, it is also necessary to focus on the usefulness of the released data. Simmons and Berger, 2016 developed a differentially private method for finding SNPs which are highly related with disease. This method also considers the output accuracy and computational efficiency, which have been cited as drawbacks. In our study, we also discuss the utility of differentially private χ^2-statistics and p-values.

There have also been studies on new statistics suitable for GWAS data. Ghodsi *et al.*, 2016 proposed a method for the Cochran-Armitage's trend test that is suitable for GWAS. Currently, there are not many papers that use p-values in the Cochran-Armitage's trend test, and they proposed to make fewer assumptions in the test. Subsequently, Wang *et al.*, 2019 presented an entirely new statistic that takes into account population stratification concerns in GWAS. In this paper, the traditional trend test approach is considered, but we need to discuss more publishable statistics in the future. For the Fisher's exact test, Poon *et al.*, 2018 proposed an algorithm that applies privacy protection techniques based on cryptographic theory. In our study, we used the concept of differential privacy to guarantee privacy, but it may be worthwhile to investigate other privacy protection based on cryptosystems and their effectiveness in protecting the privacy of statistics.

S2.2 Differential Privacy for Genomic Data

Despite the fact that the concept of differential privacy has received much attention in various fields since its inception in the field of cryptography, to the best of our knowledge, no practical way of setting the value of ϵ (i.e. privacy level) has been established. So far, challenges to this problem have been proposed, and it is hoped that these will be developed to select a more appropriate parameter for releasing of genomic data.

Firstly, Lee and Clifton, 2011 demonstrated the challenge of differential privacy parameter ϵ's impact on actual disclosure risk. They showed that the appropriate value of epsilon can be suppressed if the risk of being identified is known in advance. This means that the challenge of assessing this risk with certainty at the time of release of genomic data needs to be addressed in the future. Subsequently, Hsu *et al.*, 2014 developed a model on the balance of interests between data analysts and those who wish to participate in the data, and proposed to determine the value of ϵ on the basis of this model. However, the shortcomings of this model include the inability to determine an upper bound on the harm caused by privacy leaks and the fact that it is not clear whether the model satisfies the original concept of differential privacy for individuals. In addition, the number of parameters has been increased compared to the original concept, which may be influenced by the subject of privacy protection.

We propose appropriate values of ϵ by calculating the values of f-measure through simulation studies. In the future, it may be necessary to consider how much larger the value of ϵ can be.

S3 Methods

In this section, we give detailed proofs for theoretical guarantees of our methods for releasing ϵ-differentially private statistics. In GWAS, the number of the case and that of the control are generally set close to each other, so we assume that the total number of individuals is denoted by N, and that there are $N/2$ cases and $N/2$ controls. We set $N \geq 100$ for sake of simplicity in this work since GWAS usually considers thousands to millions of individuals. We also assume that all margins of contingency tables are positive, because GWAS generally removes SNPs with a MAF smaller than 0.05.

S3.1 ϵ-differentially Private Statistics for Chi-squared Test

S3.1.1 Case 1: 3×2 contingency table

Theorem S1. *The sensitivity of* $\log_{10}(p\text{-values})$ *obtained from the* χ^2*-statistic for genotype frequency comparisons based on a* 3×2 *contingency table, in which the margins are positive and the number of the case and the control are both* $N/2$*, is* $\log_{10}(\mathrm{e}) \cdot \frac{2N}{N+2}$.

Proof. Let x be the χ^2-statistic obtained from a 3×2 contingency table. The p-value corresponding to x is $\mathrm{e}^{-\frac{x}{2}}$, and the base 10 logarithm of the value is

$$-\frac{x}{2} \cdot \log_{10}(\mathrm{e}).$$

From Fienberg *et al.*, 2011, the sensitivity of the χ^2-statistics is $\frac{4N}{N+2}$. Therefore, the sensitivity of $\log_{10}(p\text{-values})$ is

$$\left| -\frac{1}{2} \log(\mathrm{e}) \cdot \frac{4N}{N+2} \right| = \log_{10}(\mathrm{e}) \cdot \frac{2N}{N+2}.$$

□

S3.1.2 Case 2: 2×2 contingency table

Theorem S2. *The sensitivity of the* χ^2*-statistics for allele frequency comparisons based on a* 2×2 *contingency table, in which the margins are positive and the number of the case and the control are both* N*, is* $\frac{8N}{N+2}$.

Proof. We consider the following 2×2 contingency table:

		Disease Status		Total
		0	1	
Allele	0	a	$m-a$	m
	1	$N-a$	$N-m+a$	$2N-m$
Total		N	N	$2N$

with $a \geq 0, m \geq 3, a \leq m, a \leq N, m \leq 2N-3$, and $m-a \leq N$. The reason for $m \geq 3$ and $m \leq 2N-3$ is that the 2×2 tables above corresponds to a 3×2 contingency table with positive margins, which is used for genotype frequency comparisons. The χ^2-statistic based on this table can be expressed as a function

$$\chi^2 : \mathcal{D} \longrightarrow \mathbb{R}_{\geq 0},$$

where $\mathcal{D} = \{(a, m) \in \mathbb{N} \mid a \geq 0, m \geq 3, a \leq m, a \leq N, m \leq 2N-3, m-a \leq N\}$.

Then, we consider the values of $(a, m) \in \mathcal{D} \cap \{a \geq 2, m \geq 5, m \leq 2N-3\}$, which maximize

$$\left|\chi^2(a, m) - \chi^2(a-2, m-2)\right|. \tag{1}$$

Now we can only consider the case of $m \leq N$, since

$$\left|\chi^2(a, m) - \chi^2(a-2, m-2)\right|$$
$$= \left|\chi^2(N-a+2, 2N-m+2) - \chi^2(N-a, 2N-m)\right|.$$

First note that

$$\chi^2(a, m) = \frac{(2a-m)^2}{m} + \frac{(2a-m)^2}{2N-m} = \frac{2N(2a-m)^2}{m(2N-m)},$$

We then compute the directional derivative of $\chi^2(a, m)$ in direction $(-1, -1)$, which is given by

$$-\frac{4N(2a-m)}{m(2N-m)} - \frac{4N(m-N)(2a-m)^2}{m^2(2N-m)^2}. \tag{2}$$

Here, we calculate the minimum and maximum values of (2).

(i) Minimum values.

We first prove that $\frac{2a-m}{m(2N-m)}$ takes the maximum value when $(a, m) = (N, N)$. With fixing the value m, the range of values that a can take is $[1, m]$. Therefore, it takes the maximum value when $a = m$. And since the range of possible values of m is $[2, N]$, the maximum value is taken when $(a, m) = (N, N)$.

In addition, $2a - m$ takes the maximum value N when $(a, m) = (N, N)$, and $m - N$ takes the maximum value when $m = N$. Therefore, (2) is minimized by the values $(a, m) = (N, N)$.

(ii) Maximum values.

Since $\frac{(2a-m)^2}{m^2(2N-m)^2} > 0$, the second term of (2) takes the maximum value 0 when $m = N$. We then think about the minimum value of $\frac{(2a-m)}{m(2N-m)}$. With fixing the value of m, it takes the minimum value when $a = 2$. And

$$\frac{2a-m}{m(2N-m)} = \frac{4-m}{m(2N-m)} = \frac{4}{m(2N-m)} - \frac{1}{2N-m},$$

where $a = 2$. Since $\frac{4}{m(2N-m)}$ is minimized and $\frac{1}{2N-m}$ is maximized by the value $m = N$, the first term of (2) takes the maximum value when $(a, m) = (2, m)$. Therefore, (2) is maximized by the values $(a, m) = (2, N)$.

From the above (i) and (ii), (1) can be maximized when $(a, m) = (N, N)$ or when $(a, m) = (2, N)$.

$$|\chi^2(N, N) - \chi^2(N-2, N-2)| = \frac{8N}{N+2},$$

$$|\chi^2(2, N) - \chi^2(0, N-2)| = \frac{8(N^2-8)}{N(N+2)}.$$

Consequently, the sensitivity is $\frac{8N}{N+2}$. □

Theorem S3. *The sensitivity of the p-values obtained from the* χ^2*-statistic for allele frequency comparisons based on a* 2×2 *contingency table, in which the margins are positive and the number of the case and the control are both* N*, is*

$$\frac{1}{\sqrt{2\pi}} \int_0^{\frac{N}{N-2}} x^{-\frac{1}{2}} \cdot \mathrm{e}^{-\frac{x}{2}} \, \mathrm{d}x.$$

Proof. We consider the same $2\times$ contingency table as in Theorem S2. Then the p-values can be viewed as a function

$$p : \mathcal{D} \longrightarrow \mathbb{R}_{\geq 0},$$

where $\mathcal{D} = \{(a, m) \in \mathbb{N} \mid a \geq 0, m \geq 3, a \leq m, a \leq N, m \leq 2N-3, m-a \leq N\}$. We consider maximizing

$$|p(a, m) - p(a-2, m-2)|, \tag{3}$$

where $(a, m) \in \mathcal{D} \cap \{a \geq 2, m \geq 5, m \leq 2N-3\}$. Similar to Theorem S1, we discuss only the case of $m \leq N$.

First note that

$$p(a,m) = \frac{1}{\sqrt{2\pi}} \int_{\chi^2(a,m)}^{\infty} x^{-\frac{1}{2}} \cdot \mathrm{e}^{-\frac{x}{2}} \mathrm{d}x.$$

$$\chi^2(a,m) = \frac{2N(2a-m)^2}{m(2N-m)},$$

Let

$$f(a,m) = \int_{\chi^2(a,m)}^{\infty} x^{-\frac{1}{2}} \cdot \mathrm{e}^{-\frac{x}{2}} \mathrm{d}x.$$

The absolute value of the directional derivative of $f(a,m)$ in direction $(-1,-1)$ is

$$\left|\frac{\partial}{\partial a}\chi^2(a,m) + \frac{\partial}{\partial m}\chi^2(a,m)\right| \cdot \{\chi^2(a,m)\}^{-\frac{1}{2}} \cdot \mathrm{e}^{-\frac{\chi^2(a,m)}{2}}$$
$$= \sqrt{8N} \cdot \left|\frac{(N-m)(2a-m) - m(2N-m)}{(\sqrt{m(2N-3)})^3}\right| \cdot \mathrm{e}^{-\frac{\chi^2(a,m)}{2}}. \quad (4)$$

We then let

$$g(a,m) = \frac{(N-m)(2a-m) - m(2N-m)}{(\sqrt{m(2N-3)})^3}.$$

Since $N - m \geq 0$, $a \leq m$,

$$\begin{aligned} & (N-m)(2a-m) - m(2N-m) \\ < \ & (N-m)(2m-m) - m(2N-m) \\ = \ & -Nm < 0. \end{aligned}$$

Then we find the minimum value of $g(a,m)$. With fixing the value of m, $g(a,m)$ is minimized when $a = 2$ and

$$g(2,m) = \frac{2m^2 - (4+3N)m + 4N}{(\sqrt{m(2N-m)})^3}.$$

Then

$$\begin{aligned} & \frac{\mathrm{d}}{\mathrm{d}m} g(2,m) \\ = \ & \frac{2m^3 - (8+4N)m^2 + (3N^2+16N)m - 12N^2}{\{m(2N-m)\}^{\frac{5}{2}}}, \end{aligned}$$

and let

$$h(m) = 2m^3 - (8+4N)m^2 + (3N^2+16N)m - 12N^2.$$

Here,

$$\frac{\mathrm{d}}{\mathrm{d}m} h(m) = 6m^2 - 2(8+4N)m + (3N^2+16N),$$

and

$$(8+4N)^2 - 6(3N^2+16N) = -2N^2 - 32N + 64 < 0.$$

Therefore, $h(m)$ monotonically increases. From this and $h(5) = 3N^2 - 20N + 50 > 0$, we can say $h(m) > 0$, i.e.,

$$\frac{\mathrm{d}}{\mathrm{d}m} g(2,m) > 0.$$

Thus, $g(2,m)$ takes the minimum value when $m = 5$.

As for the maximum value of $\mathrm{e}^{-\frac{\chi^2(a,m)}{2}}$, it is reached when $\chi^2(a,m) = 0$, i.e., when $m = 2a$.

From the above the value of (4) takes the maximum value either when $(a,m) = (2,5)$, or when $(a,m) = (3,6)$. Since

$$\int_{\frac{2N}{5(2N-5)}}^{\frac{6N}{2N-3}} x^{-\frac{1}{2}} \cdot \mathrm{e}^{-\frac{x}{2}} \, \mathrm{d}x < \int_0^{\frac{N}{N-2}} x^{-\frac{1}{2}} \cdot \mathrm{e}^{-\frac{x}{2}} \, \mathrm{d}x$$

the value of (3) is maximized when $(a,m) = (3,6)$.

Consequently, the sensitivity of p-values is

$$|p(3,6) - p(1,4)| = \frac{1}{\sqrt{2\pi}} \int_0^{\frac{N}{N-2}} x^{-\frac{1}{2}} \cdot \mathrm{e}^{-\frac{x}{2}} \, \mathrm{d}x.$$

□

Theorem S4. *The sensitivity of* $\log_{10}$*(p-values) obtained from the* χ^2*-statistic for allele frequency comparisons based on a* 2×2 *contingency table in which the margins are positive and the number of the case and the control are both* N*, is less than* 2.33.

Proof. The p-value corresponding to the χ^2-statistic x is

$$\frac{1}{\sqrt{2\pi}} \int_x^{\infty} x^{-\frac{1}{2}} \cdot \mathrm{e}^{-\frac{x}{2}} \mathrm{d}x,$$

and we let

$$f(x) = \log_{10}\left(\int_x^{\infty} x^{-\frac{1}{2}} \cdot \mathrm{e}^{-\frac{x}{2}} \mathrm{d}x\right).$$

Since the sensitivity of the χ^2-statistics is $\frac{8N}{N+2} < 8$ [$\because$ Theorem S1], that of $\log_{10}$(p-values) is less than the maximum value of $f(x) - f(x+8)$. Here,

$$f(x) - f(x+8) = \log_{10}\left(\frac{\int_x^{\infty} x^{-\frac{1}{2}} \cdot \mathrm{e}^{-\frac{x}{2}} \mathrm{d}x}{\int_{x+8}^{\infty} x^{-\frac{1}{2}} \cdot \mathrm{e}^{-\frac{x}{2}} \mathrm{d}x}\right),$$

and we let

$$g(x) = \frac{\int_x^{\infty} x^{-\frac{1}{2}} \cdot \mathrm{e}^{-\frac{x}{2}} \mathrm{d}x}{\int_{x+8}^{\infty} x^{-\frac{1}{2}} \cdot \mathrm{e}^{-\frac{x}{2}} \mathrm{d}x}.$$

Here, from the outline of the graph of the chi-squared distribution with 1 degree of freedom,

$$\frac{\int_x^{\infty} x^{-\frac{1}{2}} \mathrm{e}^{-\frac{x}{2}} \mathrm{d}x}{\int_{x+8}^{\infty} x^{-\frac{1}{2}} \mathrm{e}^{-\frac{x}{2}} \mathrm{d}x} < \frac{x^{-\frac{1}{2}} \mathrm{e}^{-\frac{x}{2}}}{(x+8)^{-\frac{1}{2}} \mathrm{e}^{-\frac{x+8}{2}}}$$

Therefore,

$$\begin{aligned} & \left(\int_{x+8}^{\infty} x^{-\frac{1}{2}} \mathrm{e}^{-\frac{x}{2}} \mathrm{d}x\right)^2 \cdot g'(x) \\ = \ & -x^{-\frac{1}{2}} \mathrm{e}^{-\frac{x}{2}} \left(\int_{x+8}^{\infty} x^{-\frac{1}{2}} \mathrm{e}^{-\frac{x}{2}} \mathrm{d}x\right) \\ & +(x+8)^{-\frac{1}{2}} \mathrm{e}^{-\frac{x+8}{2}} \left(\int_x^{\infty} x^{-\frac{1}{2}} \mathrm{e}^{-\frac{x}{2}} \mathrm{d}x\right) \\ < \ & 0 \end{aligned}$$

Thus, $g'(x) < 0$, which means that $g(x)$ and $f(x) - f(x+8)$ take the maximum values when $x = 0$. Since $f(0) - f(8) < 2.33$, the sensitivity of $\log_{10}$(p-values) is less than 2.33. □

S3.2 ϵ-differentially Private p-values for Fisher's Exact Test

S3.2.1 Case 1: 2×2 contingency table

Theorem S5. *The sensitivity of the Fisher's exact test p-values for allele frequency comparisons based on a 2×2 contingency table, in which the margins are positive and the number of the case and the control are both N, is $\frac{N(7N-3)}{8(2N-1)(2N-3)}$.*

Proof. We consider the same 2×2 contingency table as in Theorem S2. The p-value of the Fisher's exact test obtained from this table is

$$p = \frac{{}_N\mathrm{C}_a \cdot {}_N\mathrm{C}_{m-a}}{{}_{2N}\mathrm{C}_m}.$$

Then we think about the maximum value of

$$\left| \frac{{}_N\mathrm{C}_a \cdot {}_N\mathrm{C}_{m-a}}{{}_{2N}\mathrm{C}_m} - \frac{{}_N\mathrm{C}_{a-2} \cdot {}_N\mathrm{C}_{m-a}}{{}_{2N}\mathrm{C}_{m-2}} \right|, \tag{5}$$

where $a \geq 2$, $m \geq 5$, and $m \leq 2N-3$. Let

$$f(a,m) = \frac{{}_N\mathrm{C}_a \cdot {}_N\mathrm{C}_{m-a}}{{}_{2N}\mathrm{C}_m}.$$

Similar to Theorem S2, we only consider when $a \leq N/2$. First, we prove $f(a,m) > f(a,m+1)$ when $m \geq 2a$.

$$\begin{aligned} f(a,m+1) &= \frac{{}_N\mathrm{C}_a \cdot {}_N\mathrm{C}_{m-a+1}}{{}_{2N}\mathrm{C}_{m+1}} \\ &= \frac{m+1}{2N-m} \cdot \frac{N-m+1}{m-a+1} \cdot f(a,m), \end{aligned}$$

$$\begin{aligned} & (2N-m)(m-a+1) - (m+1)(N-m+a) \\ = \; & N(m-2a+1) - a > 0. \; [\because m \geq 2a] \end{aligned}$$

Thus, when $m \geq 2a$, $f(a,m) > f(a,m+1)$.

Next, we show $f(a,2a) > f(a+1, 2(a+1))$ when $a < N/2$.

$$\begin{aligned} & f(a+1, 2(a+1)) \\ = \; & \frac{{}_N\mathrm{C}_{a+1} \cdot {}_N\mathrm{C}_{a+1}}{{}_{2N}\mathrm{C}_{2(a+1)}} \\ = \; & \frac{(2a+2)(2a+1)}{(2N-2a)(2N-2a-1)} \cdot \frac{N-a}{a+1} \cdot \frac{N-a}{a+1} \cdot f(a,2a) \\ = \; & \frac{(2a+1)(N-a)}{(2N-2a-1)(a+1)} \cdot f(a,2a), \end{aligned}$$

$$\begin{aligned} (2N-2a-1)(a+1) - (2a+1)(N-a) &= N-2a-1 \\ &> 0. \; [\because a < N/2] \end{aligned}$$

Thus, when $a < N/2$, $f(a,2a) > f(a+1, 2(a+1))$.

From now on, we prove that the maximum value of (5) is

$$\frac{N(7N-3)}{8(2N-1)(2N-3)}.$$

(i) $a = 2$.

$$\begin{aligned} & |f(2,5) - f(0,3)| \\ = \; & \frac{3N^2+2N-6}{4(2N-1)(2N-3)} < \frac{N(7N-3)}{8(2N-1)(2N-3)}. \\ & |f(2,6) - f(0,4)| \\ = \; & \frac{(N-3)(11N^2+3N-20)}{8(2N-1)(2N-3)(2N-5)} < \frac{N(7N-3)}{8(2N-1)(2N-3)}. \end{aligned}$$

When $m \geq 7$,

$$\begin{aligned} f(2,m) &\leq f(2,7) \\ &= \frac{21N(N-1)(N-4)}{16(2N-1)(2N-3)(2N-5)} \\ &< \frac{N(7N-3)}{8(2N-1)(2N-3)}. \\ f(2,m) &> f(0,m-2) > 0. \end{aligned}$$

Therefore,

$$|f(2,m) - f(0,m-2)| < \frac{N(7N-3)}{8(2N-1)(2N-3)}.$$

(ii) $a = 3$.

As in the case of $a = 2$, when $m \leq 8$,

$$|f(3,m) - f(1,m-2)| \leq \frac{N(7N-3)}{8(2N-1)(2N-3)}.$$

When $m \geq 9$,

$$\begin{aligned} f(3,m) &\leq f(3,9) \\ &= \frac{21N(N-1)(N-2)(N-5)}{8(2N-1)(2N-3)(2N-5)(2N-7)} \\ &< \frac{N(7N-3)}{8(2N-1)(2N-3)}. \\ f(3,m) &> f(1,m-2) > 0. \end{aligned}$$

Therefore,

$$|f(3,m) - f(1,m-2)| < \frac{N(7N-3)}{8(2N-1)(2N-3)}.$$

(iii) $4 \leq a \leq 8$.

As in the case of $a = 2$ and $a = 3$, we can easily prove

$$|f(a,m) - f(a-2,m-2)| \leq \frac{N(7N-3)}{8(2N-1)(2N-3)}$$

with equality if and only if $(a,m) = (4,5)$.

(iv) $9 \leq a \leq N/2$.

We can see

$$f(7,14) < \frac{N(7N-3)}{8(2N-1)(2N-3)}.$$

Thus, when $m < a$,

$$\begin{aligned} |f(a,m) - f(a-2,m-2)| &= f(a-2,m-2) - f(a,m) \\ &< f(a-2, 2(a-2)) \\ &\leq f(7,14) \\ &< \frac{N(7N-3)}{8(2N-1)(2N-3)}. \end{aligned}$$

When $m \geq a$,

$$\begin{aligned} |f(a,m) - f(a-2,m-2)| &= f(a,m) - f(a-2,m-2) \\ &< f(a,2a) \\ &< f(7,14) \\ &< \frac{N(7N-3)}{8(2N-1)(2N-3)}. \end{aligned}$$

From the above, the sensitivity we seek is

$$\frac{N(7N-3)}{8(2N-1)(2N-3)}.$$

□

Theorem S6. *The sensitivity of* $\log_{10}(p\text{-values})$ *obtained from the Fisher's exact test for allele frequency comparisons based on a* 2×2 *contingency table, in which the margins are positive and the number of the case and the control are both N, is* $\log_{10}\left(\frac{1}{2}(N+1)(N+2)\right)$.

Proof. We consider the same 2×2 contingency table as in Theorem S2. The p-value of the Fisher's exact test obtained from the table is

$$\begin{aligned} p &= \frac{{}_N\mathrm{C}_a \cdot {}_N\mathrm{C}_{m-a}}{{}_{2N}\mathrm{C}_m} \\ &= \frac{N! \cdot N! \cdot m! \cdot (2N-m)!}{(2N)! \cdot a! \cdot (m-a)! \cdot (N-a)! \cdot (N-m+a)!}. \end{aligned}$$

Now we let $f(a, m)$ be the right side of this equation, then we think about the maximum value of

$$\begin{aligned} &\left|\log_{10}\left(f(a,m)\right) - \log_{10}\left(f(a-2,m-2)\right)\right| \\ = &\left|\log_{10}\left(\frac{f(a,m)}{f(a-2,m-2)}\right)\right|. \end{aligned} \quad (6)$$

Below, we find the maximum value of

$$\frac{f(a,m)}{f(a-2,m-2)} = \frac{m(m-1)(N-a+1)(N-a+2)}{a(a-1)(2N-m+1)(2N-m+2)}. \quad (7)$$

The smaller the value of a and the larger the value of m, the larger (7) takes, so we can consider the case of $m - a = N$. Then

$$\begin{aligned} (5) &= \frac{(N+a)(N+a-1)}{a(a-1)} \\ &= \frac{1}{a(a-1)}N^2 + \frac{2a-1}{a(a-1)}N + 1. \end{aligned}$$

Therefore, (7) is maximized when $(a, m) = (2, N+2)$, and the maximum value of (6) is

$$\log_{10}\left(\frac{1}{2}(N+1)(N+2)\right).$$

□

S3.2.2 Case 2: 3 × 2 contingency table

Theorem S7. *The sensitivity of* $\log_{10}(p\text{-values})$ *obtained from the Fisher's exact test for genotype frequency comparisons based on a* 3×2 *contingency table, in which the margins are positive and the number of the case and the control are both* $N/2$*, is* $\log_{10}\left(\frac{N}{2}+1\right)$.

Proof. We consider the following 3×2 contingency table:

		Disease Status		Total
		0	1	
Genotype	0	a	$m-a$	m
	1	b	$n-b$	n
	2	$N/2-a-b$	$N/2-m-n+a+b$	$N-m-n$
Total		$N/2$	$N/2$	N

with $a \geq 0, b \geq 0, m > 0, n > 0, a \leq m, b \leq n, a+b \leq N/2, m+n < N$, and $m+n-a-b \leq N/2$. The p-value of the Fisher's exact test obtained from this table is

$$p = \frac{{}_m\mathrm{C}_a \cdot {}_n\mathrm{C}_b \cdot {}_{N-m-n}\mathrm{C}_{N/2-a-b}}{{}_N\mathrm{C}_{N/2}}.$$

Now we let $f(a, b, m, n)$ be the right side of this equation. Then we think about the maximum value of

$$\begin{aligned} &\left|\log_{10}\left(f(a,b,m,n)\right) - \log_{10}\left(f(a-1,b+1,m-1,n+1)\right)\right| \\ = &\left|\log_{10}\left(\frac{f(a,b,m,n)}{f(a-1,b+1,m-1,n+1)}\right)\right|. \end{aligned}$$

Here,

$$\frac{f(a,b,m,n)}{f(a-1,b+1,m-1,n+1)} = \frac{m(b+1)}{a(n+1)}, \quad (8)$$

and since $b \leq n$ and $m \leq a + N/2$, (8) takes the maximum value $\frac{N}{2}+1$ when $a = 1, m = N/2+1$, and $b = n$. Therefore, the sensitivity of $\log_{10}(p\text{-values})$ is

$$\log_{10}\left(\frac{N}{2}+1\right).$$

□

S3.3 ϵ-differentially Private Statistics for Cochran-Armitage's Trend Test

Theorem S8. *The sensitivity of the* χ^2*-statistics of the Cochran-Armitage's trend test based on a* 3×2 *contingency table, in which the margins are positive and the number of the case and the control are both* $N/2$*, is* $\frac{16N(N^2+6N+4)}{(N+18)(N^2+8N-4)}$.

Proof. We consider the same 3×2 contingency table as in Theorem S7. The χ^2-statistic of the Cochran-Armitage's trend test obtained from the table is

$$T = \frac{N(2m+n-2(2a+b))^2}{4Nm+Nn-(2m+n)^2}.$$

This statistic can be expressed as a function

$$\chi^2 : \mathcal{D} \longrightarrow \mathbb{R}_{\geq 0},$$

where $\mathcal{D} = \{(a,b,m,n) \in \mathbb{N} \mid a \geq 0, b \geq 0, m > 0, n > 0, a \leq m, b \leq n, a+b \leq N/2, m+n < N, m+n-a-b \leq N/2\}$. Now we consider maximizing

$$\left|\chi^2(a-1,b,m-1,n) - \chi^2(a,b,m,n)\right| \quad (9)$$

where $(a,b,m,n) \in \mathcal{D} \cap \{a \geq 1, m \geq 2\}$. Similar to Theorem S1., we can consider only the case of $2m + n \leq N + 1$.

First, we calculate the maximum value of

$$\chi^2(a-1,b,m-1,n) - \chi^2(a,b,m,n). \quad (10)$$

Here we can calculate

$$\chi^2(a-1,b,m-1,n) - \chi^2(a,b,m,n) = \frac{N\{f(a,b,m,n)\}}{\{4Nm+Nn-4N-(2m+n-2)^2\}\{4Nm+Nn-(2m+n)^2\}},$$

where

$$\begin{aligned} f(a,b,m,n) &= \{2m+n-2(2a+b)\}^2\{4N-4(2m+n)+4\} \\ &+ 4\{2m+n-2(2a+b)\}\{4Nm+Nn-(2m+n)^2\} \\ &+ 4\{4Nm+Nn-(2m+n)^2\}. \end{aligned}$$

Then

$$\frac{\partial}{\partial a} f(a,b,m,n)$$
$$= -16[8Nm + 3Nn - 3(2m+n)^2 + 2(2m+n)$$
$$- 4(2a+b)\{N - (2m+n) + 1\}].$$

Since $2m + n \le N + 1$,

$$\frac{\partial^2}{\partial a^2} f(a,b,m,n) = 128\{N - (2m+n) + 1\} > 0.$$

Therefore, $f(a)$ takes the maximum value when $a = 1$ or when $a = \min\{N/2 - b, m\}$. Similarly, the maximum value of (8) is taken when $b = 0$ or when $b = \min\{N/2 - a, n\}$. Thus, (8) can be maximized when $(a,b) = (1,0), (1, N/2 - 1), (1,n), (N/2, 0), (N/2 - n, n), (m, 0), (m, n), (m, N/2 - m)$.

When $(a,b) = (1,0)$, the maximum value

$$\frac{16N(N^2 + 6N + 4)}{(N^2 + 8N - 4)(N + 18)}$$

is taken at the case of $(a,b,m,n) = (1,0,2,N/2 - 1)$.

When $(a,b) = (1,n)$, the maximum value

$$\frac{4N(N-4)}{N^2 - N - 1}$$

is taken at the case of $(a,b,m,n) = (1,1,N/2,1)$.

When $(a,b) = (m,0)$, the maximum value

$$\frac{16N^3(N-6)}{(N^2 + 8N - 16)(N^2 + 16N - 64)}$$

is taken at the case of $(a,b,m,n) = (2,0,2,N/2)$.

Otherwise, the value of (10) is negative. Therefore, the maximum value of (10) is

$$\frac{16N(N^2 + 6N + 4)}{(N^2 + 8N - 4)(N + 18)}$$

where $(a,b,m,n) = (1,0,2,N/2 - 1)$.

Next, we calculate the minimum value of (7). The directional derivative of $\chi^2(a,b,m,n)$ in direction $(-1,0,-1,0)$ is

$$\frac{4N\{2m + n - 2(2a+b)\}}{4Nm + Nn - (2m+n)^2} + \frac{4N\{N - (2m+n)\}\{2m + n - 2(2a+b)\}^2}{\{4Nm + Nn - (2m+n)^2\}^2}. \quad (11)$$

Then we find (a,b,m,n) which minimizes (11).

(i) $2m + n = N + 1$.

$4Nm + Nn - (2m+n)^2$ is minimized when $(m,n) = (N/2, 1)$.

Since the minimum value of $2m + n - 2(2a+b)$ is $-N + 1$ when $(a,b,m,n) = (N/2, 0, N/2, 1)$ and

$$2m + n - 2(2a+b) \le N + 1 - 2(2 \cdot 1 + 0) = N - 3,$$

the minimum value of $2m+n-2(2a+b)$ and the maximum value of $\{2m+n-2(2a+b)\}^2$ is taken when $(a,b,m,n) = (N/2, 0, N/2, 1)$.

Now we can see

$$N - (2m+n) = -1.$$

Therefore, when $(a,b,m,n) = (N/2, 0, N/2, 1)$, (4.10) takes the minimum value.

(ii) $2m + n \le N$.

The minimum value of the first term of (11) is taken when $(a,b,m,n) = (N/2, 0, N/2, 1)$, so it is greater than or equal to the minimum value when $2m + n \le N$. And the value of the second term of (10) is greater than or equal to 0.

From above, (11) is minimized when $(a,b,m,n) = (N/2, 0, N/2, 1)$. Therefore, the minimum value of (10) is

$$\chi^2(N/2 - 1, 0, N/2 - 1, 1) - \chi^2(N/2, 0, N/2, 1) = -\frac{4N(N-2)}{N^2 - N - 1}.$$

Consequently, (9) takes the maximum value

$$\frac{16N(N^2 + 6N + 4)}{(N^2 + 8N - 4)(N + 18)}$$

when $(a,b,m,n) = (1,0,2,N/2 - 1)$, and this value is the sensitivity of χ^2-statistics. □

Theorem S9. *The sensitivity of* $\log_{10}$(*p-values*) *obtained from the* χ^2-*statistic of the Cochran-Armitage's trend test based on a* 3×2 *contingency table, in which the margins are positive and the number of the case and the control are both* $N/2$, *is* $\log_{10}(\mathrm{e}) \cdot \frac{8N(N^2+6N+4)}{(N+18)(N^2+8N-4)}$.

Proof. We consider the same 3×2 contingency table as in Theorem S7. Then, The p-value corresponding to $\chi^2(a,b,m,n)$ is as follows:

$$p = \mathrm{e}^{-\frac{\chi^2(a,b,m,n)}{2}}.$$

Therefore, we find the maximum value of

$$\left| \log_{10}\left(\mathrm{e}^{-\frac{\chi^2(a-1,b,m-1,n)}{2}}\right) - \log\left(\mathrm{e}^{-\frac{\chi^2(a,b,m,n)}{2}}\right) \right|$$
$$= \frac{1}{2} \cdot \log_{10}(\mathrm{e}) \cdot \left|\chi^2(a-1,b,m-1,n) - \chi^2(a,b,m,n)\right| \quad (12)$$

The sensitivity of the χ^2-statistics is

$$\frac{16N(N^2 + 6N + 4)}{(N^2 + 8N - 4)(N + 18)},$$

so the maximum value of (12), i.e. the sensitivity of $\log_{10}$(p-values) is

$$\log_{10}(\mathrm{e}) \cdot \frac{8N(N^2 + 6N + 4)}{(N^2 + 8N - 4)(N + 18)}$$

□

Based on the sensitivity of the statistical functions shown in the above proofs, ϵ-differential privacy can be achieved by applying the Laplace mechanism to genomic datasets.

S3.4 When not an equal number of cases and controls

In this subsection, we consider sensitivity when the number of the case and that of the control are different. Firstly, we give theorems for sensitivity of $\log_{10}$(p-values) in the Fisher's exact test.

Theorem S10. *The sensitivity of* $\log_{10}$(*p-values*) *obtained from the Fisher's exact test for allele frequency comparisons based on a* 2×2 *contingency table, in which the margins are positive and the number of the case to that of the control is* $k : (1-k)$ *or* $(1-k) : k$ $(0 < k < \frac{1}{2})$, *is* $\log_{10}\left((2(1-k)N + 1)\left((1-k)N + 1\right)\right)$.

Theorem S11. *The sensitivity of* $\log_{10}$(*p-values*) *obtained from the Fisher's exact test for allele frequency comparisons based on a* 3×2 *contingency table, in which the margins are positive and the number of*

the case to that of the control is $k : (1-k)$ *or* $(1-k) : k \ \ (0 < k < \frac{1}{2})$, *is* $\log_{10}((1-k)N+1)$.

The proofs of Theorem S10 and Theorem S11 are similar to the proofs of Theorem S6 and Theorem S7, respectively. Therefore, the amount of noise to be added is not much different from the case where the number of the case is equal to that of the control, and the same approach described up to Section S3.3 could be applied.

As for the other statistics, we expect that the sensitivity is comparable to which shown up to Section S3.3. Since rigorous proofs were not shown in this study, it will be necessary to consider more detailed sensitivities which also takes into account genomic dependencies in the future.

S4 Experiments and Discussion

In this section, we discuss supporting results and discussions of our experiments. We measured the utility of the private statistics by calculating the KL divergence (Kullback and Leibler, 1951) between the original statistics and the noise-added statistics by our experiments. Based on the number of participants in a typical GWAS, we considered the cases where the number of individuals in the simulation data were $N = 1000,\ 10\,000,\ 50\,000,\ 100\,000$.

S4.1 ϵ-differentially Private Statistics for Chi-squared Test

S4.1.1 Case 2: 2 × 2 contingency table

In this supplement, we assessed the utility of our private χ^2-statistics, p-values, and $-\log_{10}(p\text{-values})$.

Firstly, in order to assess the utility of the private χ^2-statistics, we obtained the KL divergence between the original and the private χ^2-statistics. Similar to the case using a 3×2 contingency table, we created datasets with noise based on Theorem S2, and in Fig. S1 we plotted the obtained KL divergence. The value of ϵ was varied from 0.1 to 10. Fig. S1 shows that our method to release ϵ-differentially private χ^2-statistics might be useful if ϵ is greater than or equal to 5. Therefore, we consider the thresholds when the ϵ is 5, 7, and 10 in the main paper.

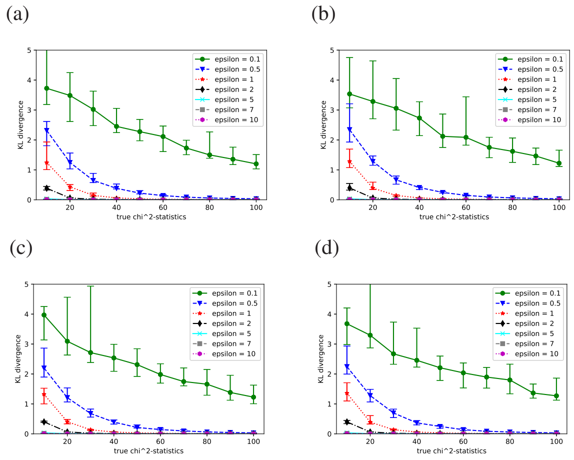

Fig. S1. KL divergence between the original and the private χ^2-statistics in chi-squared test with a 2×2 contingency table when (a) $N = 1000$, (b) $N = 10\,000$, (c) $N = 50\,000$, (d) $N = 100\,000$.

Secondly, we evaluated the utility of private p-values. As in the case of the χ^2-statistics, we calculated the KL divergence between the original p-values and the perturbed p-values based on Theorem S3. Fig. S2 shows the KL divergence obtained from our experiment. Compared to the case of χ^2-statistics, the values are quite large, indicating that private p-values are not practical. This fact is the same as in the case of the chi-squared test with a 3×2 contingency table. Therefore, it might be better to consider the base 10 logarithm of the p-values when releasing the statistics.

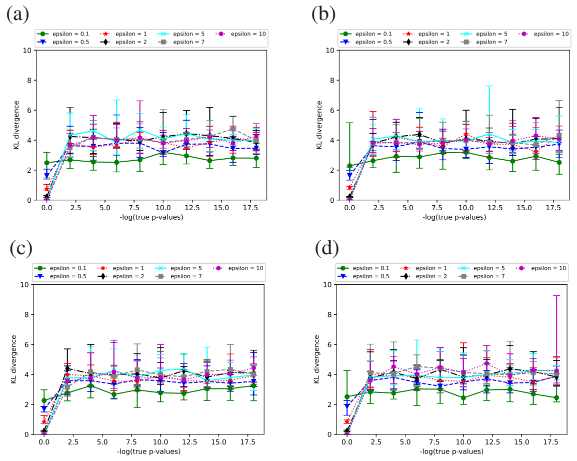

Fig. S2. KL divergence between the original and the private p-values in chi-squared test with a 2×2 contingency table when (a) $N = 1000$, (b) $N = 10\,000$, (c) $N = 50\,000$, (d) $N = 100\,000$.

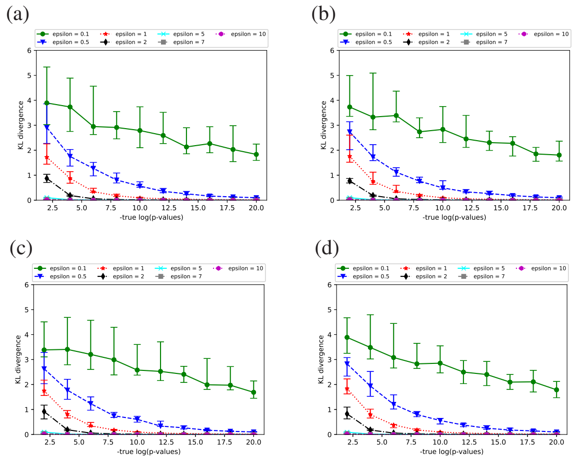

Fig. S3. KL divergence between the original and the private $-\log_{10}(p\text{-values})$ in chi-squared test with a 2×2 contingency table when (a) $N = 1000$, (b) $N = 10\,000$, (c) $N = 50\,000$, (d) $N = 100\,000$.

Finally, we assessed the practicality of our method for releasing the private $-\log_{10}(p\text{-values})$. Fig. S3 shows the KL divergence between the original values and the noise added statistics based on Theorem S4. From the figure, we consider the practical thresholds for the case of $\epsilon = 5$, 7, and 10. Same as in Case 1 in the main paper, we considered the thresholds as $-\log_{10}(p\text{-values})$, and varied thresholds from 6.0 to 9.0 in increments 0.1. For each threshold, we calculated precision, recall, and f-measure, and plotted these values in Fig. S4. When $\epsilon = 5$, these values are so small that it might be difficult to use in practice. When $\epsilon = 7$, the f-measure is maximized when threshold is 7.5, and the value is around 0.6. Although precision is less than 0.8, it might be possible to set ϵ to 7. When $\epsilon = 10$, the value of precision is also large enough that we can assure the utility of the releasing method. From the above discussion, when using ϵ-differentially

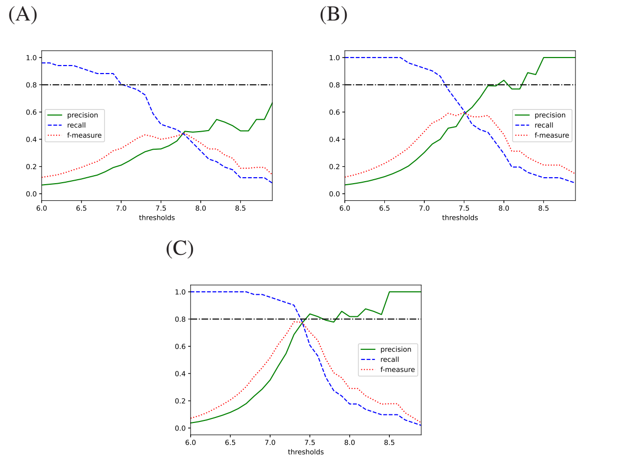

Fig. S4. Relationship between thresholds of private $-\log_{10}(p\text{-values})$ and precision, recall, and f-measure in chi-squared test with a 2×2 contingency table when (A) $\epsilon = 5$, (B) $\epsilon = 7$, (C) $\epsilon = 10$.

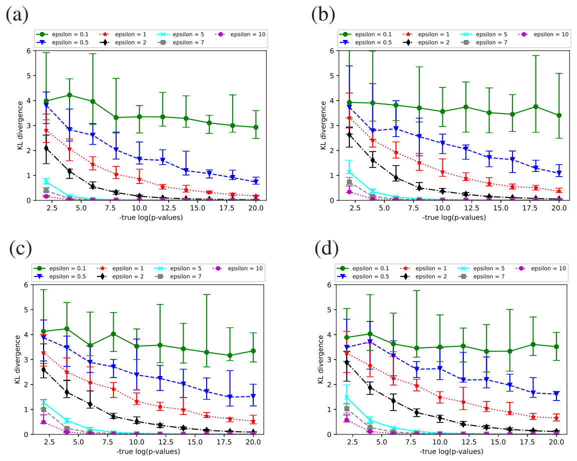

Fig. S6. KL divergence between the original and the private $-\log_{10}(p\text{-values})$ in the Fisher's exact test with a 2×2 contingency table when (a) $N = 1000$, (b) $N = 10\,000$, (c) $N = 50\,000$, (d) $N = 100\,000$.

private base 10 logarithms of the p-values in practice, the appropriate thresholds with high accuracy will be 7.5 for the case of $\epsilon = 7, 10$.

S4.2 ϵ-differentially Private p-values for Fisher's Exact Test

S4.2.1 Case 1: 2 × 2 contingency table

Firstly, we evaluated our method for releasing the p-values. As in Section 3.1, we calculated the KL divergence between the original p-values and those with noise added based on Theorem S5. In Fig. S5, we plotted the values obtained here. Similar to the case of the p-values in the chi-squared test, the KL divergence is very large regardless of the value of ϵ. The reason for this might be that the noise added is so large compared to the original p-values that the perturbed data out of the range $[0, 1]$ are needed to be adjusted to 0 or 1. This operation may lower the privacy level, and Fig. S5 suggests that the releasing method for p-values in the Fisher's exact test is useless.

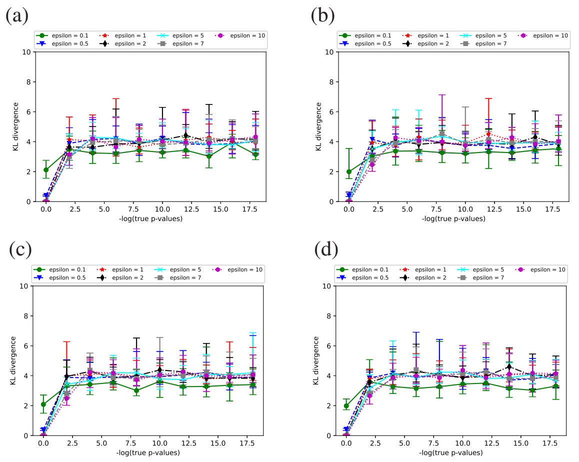

Fig. S5. KL divergence between the original and the private p-values in the Fisher's exact test with a 2×2 contingency table when (a) $N = 1000$, (b) $N = 10\,000$, (c) $N = 50\,000$, (d) $N = 100\,000$.

Next, we evaluated the method for releasing $-\log_{10}(p\text{-values})$. Fig. S6 shows the KL divergence between the original and the private $-\log_{10}(p\text{-values})$ based on Theorem S6. Fig. S6 differs from Fig. S5 in that the larger the value of ϵ, the smaller the KL divergence. This is probably due to the fact that the upper limit of values is eliminated by taking logarithms. However, even when the value of $\epsilon = 10$, the KL divergence is relatively large, suggesting that these statistics not as practical as the private χ^2-statistics in the chi-squared test.

From the above, it would be more practical to release $\log_{10}(p\text{-values})$ than p-values themselves as in the case of the chi-squared test. In the following, we considered the thresholds for using these statistics. Similar to Section 4.1, we calculated precision, recall, and f-measure. In this experiment, we considered the case of $N = 100$ and 1,000. Regarding the value of ϵ, we experimented with the case of $\epsilon = 7$ and 10. The resulting plots are shown in Fig. S7. When $N = 100$, our method could be practical if we set ϵ to 10. The maximum value of f-measure is taken when the threshold is 7.9, and this will be a proper threshold for practical use. On the other hand, when $N = 1{,}000$, the figures indicate that this method is almost impractical. The reason for this is supposed that the amount of added noise is about 1.5 times larger than that when $N = 100$.

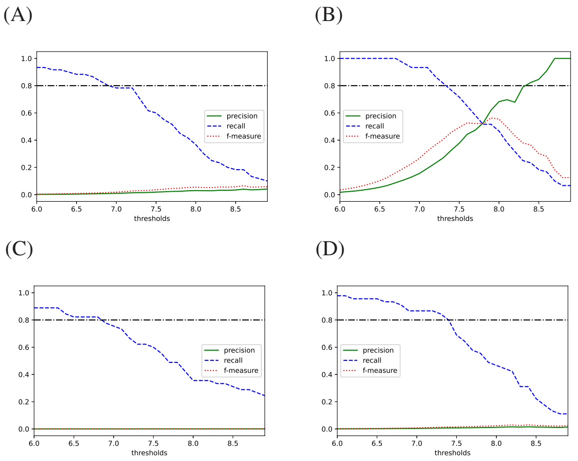

Fig. S7. Relationship between thresholds of private $-\log_{10}(p\text{-values})$ and precision, recall, and f-measure in the Fisher's exact test with a 2×2 contingency table when (A) $N = 100$, $\epsilon = 7$, (B) $N = 100$, $\epsilon = 10$, (C) $N = 1000$, $\epsilon = 7$, (D) $N = 1000$, $\epsilon = 10$.

The above experiments and discussion show that it might be possible to maintain both privacy and utility by considering the $\log_{10}(p\text{-values})$ when using and releasing private statistics in the Fisher's exact test. However, our method can be applied only when N is small and ϵ is reasonably large. Therefore, in the future, it is necessary to develop the test methods specifically for the case of N is large and to study the risk of privacy violation by increasing the value of ϵ.

S4.2.2 Case 2: 3×2 contingency table

As in the case with a 2×2 table, we evaluated the method for releasing $\log_{10}(p\text{-values})$. The KL divergence calculated from the original and the private $-\log_{10}(p\text{-values})$ is shown in Fig. S8. Here, we added noise based on Theorem S7. Compared to Fig. S6, the KL divergence is small, and the utility of this method is expected to be high.

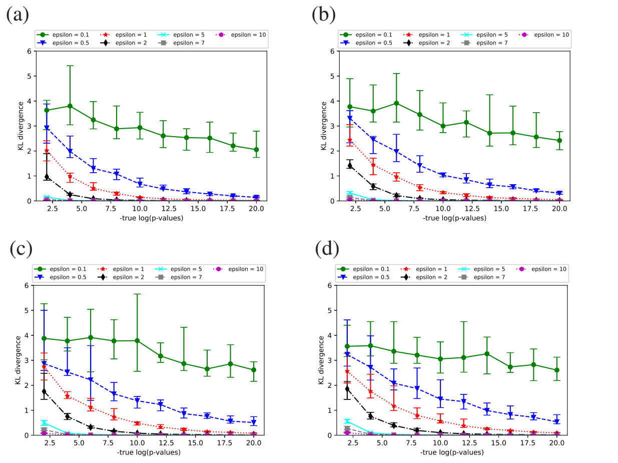

Fig. S8. KL divergence between the original and the private $-\log_{10}(p\text{-values})$ in the Fisher's exact test with a 3×2 contingency table when (a) $N = 1000$, (b) $N = 10\,000$, (c) $N = 50\,000$, (d) $N = 100\,000$.

S4.3 ϵ-differentially Private Statistics for Cochran-Armitage's Trend Test

Similar to the case of the chi-squared test, we calculated the KL divergence for the χ^2-statistics and the results are shown in Fig. S9. Here, the added noise to the original dataset is based on Theorem S8.

Next, we evaluated the method of releasing p-values in the Cochran-Armitage's trend test. We considered releasing $\log_{10}(p\text{-values})$ and adding noise based on Theorem S9. Similar to the case of the Fisher's exact test, we calculated the KL divergence between the original and private $-\log_{10}(p\text{-values})$. Fig. S10 shows these results. The sketch of this figure is almost the same as that of Fig. S9, suggesting that the same level of privacy might be achieved as when releasing the χ^2-statistics. Then, based on this figure, we evaluated the appropriate thresholds.

S4.4 Application to Real Dataset

In this subsection, we show the results of applying our method to a real dataset. The dataset we used is UKB MDD data by Coleman *et al.*, 2020 provided in LD Hub (Zheng *et al.*, 2017). The empirical p-value in this dataset is set to 5×10^{-5}, and the threshold of $-\log_{10}(p\text{-values})$ is 4.3. Since this dataset is based on 2×2 contingency tables, the private threshold is set to 4.4 from Supplementary Section 4.1.1. Then, we considered the datasets with noise described in the Methods Section, and calculated the values of precision, recall, and f-measure.

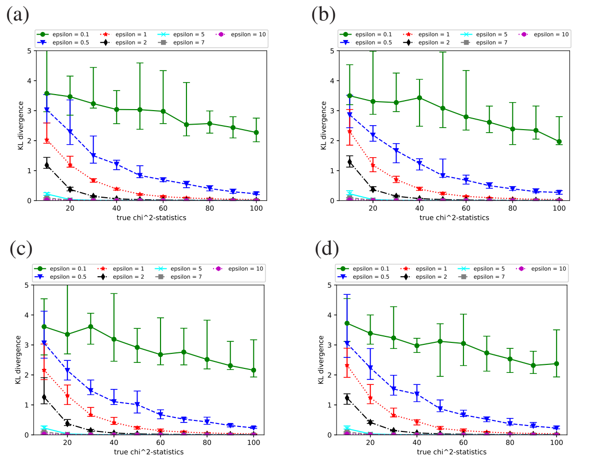

Fig. S9. KL divergence between the original and the private χ^2-statistics in the Cochran-Armitage's trend test when (a) $N = 1000$, (b) $N = 10\,000$, (c) $N = 50\,000$, (d) $N = 100\,000$.

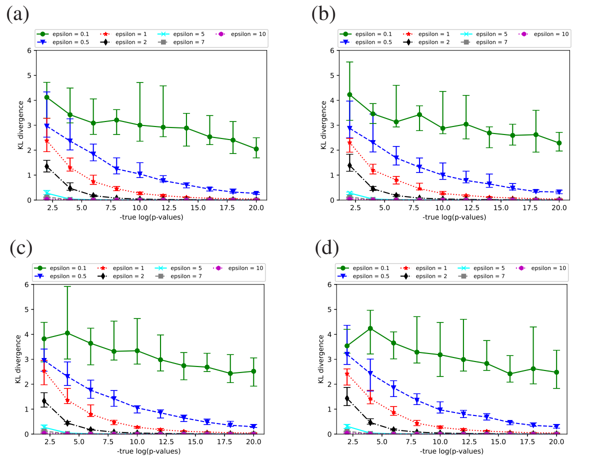

Fig. S10. KL divergence between the original and the private $-\log_{10}(p\text{-values})$ in the Cochran-Armitage's trend test when (a) $N = 1000$, (b) $N = 10\,000$, (c) $N = 50\,000$, (d) $N = 100\,000$.

(i) $\epsilon = 7$

$$\begin{aligned} Precision &= \frac{1785}{2630} = 0.679 \\ Recall &= \frac{1785}{2158} = 0.827 \\ F-measure &= 2 \times \frac{0.679 \times 0.827}{0.679 + 0.827} = 0.746 \end{aligned}$$

(ii) $\epsilon = 10$

$$\begin{aligned} Precision &= \frac{1854}{2198} = 0.843 \\ Recall &= \frac{1854}{2158} = 0.859 \\ F-measure &= 2 \times \frac{0.843 \times 0.859}{0.843 + 0.859} = 0.851 \end{aligned}$$

This results imply that our method is more practical for real data than for simulated data, i.e., we can obtain significant data with high accuracy. In the future, it is necessary to develop our methods to take dependencies among SNPs into account and apply them to more real datasets.

References

Armitage, P. (1955). Tests for linear trends in proportions and frequencies. *Biometrics*, **11**(3), 375–386.

Coleman, J. R. I. *et al.* (2020). The genetics of the mood disorder spectrum: Genome-wide association analyses of more than 185,000 cases and 439,000 controls. *Biol Psychiatry.*, **88**(2), 169–184.

Dwork, C. (2006). Differential privacy. *Michele Bugliesi, Bart Preneel, Vladimiro Sassone, and Ingo Wegener, (eds) Automata, Languages and Programming*, **4052**.

Dwork, C. *et al.* (2006). Calibrating noise to sensitivity in private data analysis. *S. Halevi and T. Rabin, (eds) Theory of Cryptography*, **3876**.

Fienberg, S. E. *et al.* (2011). Privacy preserving GWAS data sharing. *IEEE 11th International Conference on Data Mining Workshops*, pages 628–635.

Fisher, R. A. (1935). The design of experiments. *Oliver and Boyd*.

Ghodsi, M. *et al.* (2016). An enhanced version of Cochran-Armitage trend test for genome-wide association studies. *Meta Gene*, **9**, 225–229.

Hsu, J. *et al.* (2014). Differential privacy: An economic method for choosing epsilon. *2014 IEEE Computer Security Foundations Symposium*, pages 398–410.

Johnson, A. and Shmatikov, V. (2015). Privacy-preserving data exploration in genome-wide association studies. *KDD*, pages 1079–1087.

Kosheleva, O. and Kreinovich, V. (2017). Why deep learning methods use KL divergence instead of least squares: A possible pedagogical explanation. *Mathematics*.

Kullback, S. and Leibler, R. A. (1951). On information and sufficiency. *Ann. Math. Statist.*, **22**, 79–86.

Lee, J. and Clifton, C. (2011). How much is enough? Choosing ϵ for differential privacy. *X. Lai, J. Zhou, and H. Li, (eds), Information Security*, **7001**, 325–340.

Poon, A. *et al.* (2018). Privacy preserving Fisher's exact test on genomic data. *2018 IEEE International Conference on Big Data (Big Data)*, pages 2546–2553.

Simmons, S. and Berger, B. (2016). Realizing privacy preserving genome-wide association studies. *Bioinformatics*, **32**(9), 1293–1300.

Wang, Y. *et al.* (2019). Robust reference powered association test for genome-wide association studies. *Front Genet.*, **10**, 319.

Zheng, J. *et al.* (2017). LD Hub: a centralized database and web interface to perform LD score regression that maximizes the potential of summary level GWAS data for SNP heritability and genetic correlation analysis. *Bioinformatics*, **33**(2), 272–279.
